# Single-Cell Spatial–Temporal Analysis of *ZNF451* in Mediating Drug Resistance and CD8^+^ T Cell Dysfunction

**DOI:** 10.34133/research.0530

**Published:** 2024-11-12

**Authors:** Ning Tang, Woding Deng, Yupeng Wu, Zhixuan Deng, Xin Wu, Jianbin Xiong, Qiangqiang Zhao

**Affiliations:** ^1^ Department of Orthopaedics, Third Xiangya Hospital, Central South University, Changsha, Hunan, China.; ^2^ Department of Orthopaedics , Liuzhou Municipal Liutie Central Hospital, Liuzhou, Guangxi, China.; ^3^ Xiangya School of Medicine, Central South University, Changsha, Hunan, China.; ^4^ Department of Spine Surgery, First Affiliated Hospital of University of South China, Hengyang, Hunan, China.; ^5^ Institute of Cell Biology, Hengyang Medical School, University of South China, Hengyang, Hunan, China.; ^6^ Department of Spine Surgery, Third Xiangya Hospital, Central South University, Changsha, Hunan, China.; ^7^ Department of Hematology, Liuzhou People’s Hospital affiliated to Guangxi Medical University, Liuzhou, Guangxi, China.; ^8^ Department of Hematology, The Qinghai Provincial People’s Hospital, Xining, Qinghai, China.

## Abstract

Cisplatin is widely used to treat osteosarcoma, but recurrent cases often develop resistance, allowing the disease to progress and complicating clinical management. This study aimed to elucidate the immune microenvironment of osteosarcoma, providing insights into the mechanisms of recurrence and identifying potential therapeutic strategies. By analyzing multiple single-cell and bulk RNA-sequencing datasets, we discovered that the SUMOylation-related gene *ZNF451* promotes osteosarcoma recurrence and alters its immune microenvironment. *ZNF451* was found to importantly enhance the growth, migration, and invasion of resistant cells while also reducing their sensitivity to cisplatin and lowering their apoptosis rate. Moreover, our data indicated that *ZNF451* plays a crucial role in bone resorption and epithelial–mesenchymal transition. *ZNF451* also regulates CD8^+^ T cell function, leading to their exhaustion and transition to the CD8T.EXH state. Additionally, β-cryptoxanthin has been identified as a potential therapeutic agent that inhibits osteosarcoma progression by targeting *ZNF451*. In summary, these findings highlight the critical role of *ZNF451* in promoting osteosarcoma progression and underscore its potential as a therapeutic target and biomarker for osteosarcoma.

## Introduction

Osteosarcoma, most prevalent among children and young adults, is the most frequent primary bone cancer. However, its rarity affects the development of advanced treatments [1,2]. Since the 1980s, conventional treatment strategies, such as a combination of surgery and chemotherapy, have only modestly improved survival rates. This limited progress is largely due to the biological diversity and complexity of osteosarcoma [3,4]. Recent advances in molecular pathology have highlighted new avenues for understanding the molecular mechanism underlying osteosarcoma and identifying personalized treatment options [5]. Nonetheless, with the challenges of recurrence and metastasis, there is an imperative need for innovative therapeutic targets and strategies to enhance treatment efficacy and bolster long-term prognoses [6,7].

Addressing recurrent osteosarcoma highlights the need for an in-depth understanding of its pathogenesis and the identification of novel therapeutic targets. Although existing treatments struggle to address the disease’s heterogeneity and complexity, they offer opportunities for the development of personalized therapies tailored to specific molecular profiles across diverse patient populations [2,8]. The immune microenvironment of osteosarcoma, composed of immune cells, cytokines, and signaling molecules, offers an intriguing avenue for research [6,9,10]. Given the pivotal roles of various immune cells in tumor growth, metastasis, and response to treatment, comprehensive research on their interactions in osteosarcoma could reveal new drugs that modulate immune response [11–14]. Enhancing current treatment protocols, including surgery and chemotherapy, and innovating new strategies that manipulate specific immune pathways could greatly benefit patients with osteosarcoma.

In recent years, the rapid advancement of single-cell sequencing technology has provided an unprecedented detailed perspective for uncovering tumor cell heterogeneity and microenvironmental interactions [10,15,16]. In osteosarcoma research, several research teams have successfully constructed single-cell resolution maps of osteosarcoma cells, substantially enhancing our understanding of the disease’s complex biology [15,17,18]. Additionally, studies focusing on cellular communications within the tumor microenvironment and the identification of key genes have appreciably deepened our understanding of the role of cell signaling in osteosarcoma development [10,19,20]. Although these studies offer valuable insights into the biology and treatment challenges of osteosarcoma, research into recurrent osteosarcoma remains insufficient. Our study integrates advanced molecular and cellular biology techniques, including single-cell sequencing and bulk RNA sequencing (RNA-seq), to construct a single-cell resolution map of osteosarcoma. We focused on analyzing intercellular communication within the tumor microenvironment. By comparing the molecular characteristics of tumor tissues from patients with recurrent osteosarcoma with those of primary tumors, we aimed to further elucidate the mechanisms of intra- and intercellular communication in recurrent osteosarcoma and uncover potential therapeutic targets. Furthermore, this study identified SUMOylation-related genes closely associated with osteosarcoma, and through screening active ingredients in traditional Chinese medicine, innovative identification of potential therapeutic candidates with clinical translational value provides new scientific evidence for the development of personalized treatment and precision medicine strategies for osteosarcoma.

## Materials and Methods

### Data collection and enhancement

This study leveraged the Gene Expression Omnibus (GEO) database to select single-cell RNA (scRNA) data from 2 osteosarcoma databases (GSE152048 [15] and GSE162454 [17]), an osteosarcoma-related lymphocyte dataset (GSE198896 [18]), and 2 control bone samples (GSE169396 [21] and GSE217792 [15]). Patient information and sequencing specifics were sourced from the supplementary material of related publications. We enriched the analysis by incorporating RNA-seq data and clinical records from the TARGET-OS project, a part of the TARGET initiative. The GSE21257 dataset [22], rich in survival data, served as a validation set for model reliability. Samples without comprehensive survival or clinical information were omitted. We processed the scRNA-seq data extensively using the Seurat R package (version 4.3.0) [23]. DoubletFinder was employed to remove potential doublet cells, and genes like hemoglobin and mitochondrial genes were selectively filtered out. All gene data were normalized and subjected to principal components analysis (PCA) for dimensionality reduction. To mitigate batch variations, we utilized the “Harmony” package. The FindVariableFeatures function was applied to the refined data to pinpoint highly variable genes (HVGs). Following cell categorization, advanced reduction techniques, uniform manifold approximation and projection (UMAP) and t-distributed stochastic neighbor embedding (t-SNE), were used for further analysis of the sample data. Marker genes were identified using the CellMarker database and relevant scholarly work. Ultimately, the pseudotemporal developmental trajectories of cells were visualized via Monocle 2 and Monocle 3.

### Intercellular signaling analysis

In exploring the intricacies of intercellular communication across various cell clusters from scRNA-seq data, the CellChat toolkit (v1.6.1) [24] in R was utilized. We began by crafting a CellChat object with the “createCellChat” function, amalgamating RNA expression matrices and pertinent cell data. This was followed by incorporating interaction databases encompassing “Secreted Signaling”, “ECM-Receptor”, and “Cell-Cell Contact” to advance our signal analysis. The communication probabilities among cells were calculated using the “computeCommunProb” function. In the “selectK” procedure, our emphasis was on discerning global communication patterns, setting the nPatterns parameter to 2 to gauge both incoming and outgoing information flows.

### SUMOylation-related gene identification and survival analysis

We initially identified a subset of candidate genes at the intersection of small ubiquitin-like modifier (SUMO)-related genes and differentially expressed genes (DEGs). Utilizing the GSE21257 dataset, we conducted univariate Cox proportional hazards regression analysis on these genes to pinpoint those significantly associated with prognosis. Individual gene survival analysis was performed using R’s “survival” package. Samples were stratified into low- and high-expression groups based on predetermined expression cutoffs for each gene. Kaplan–Meier plots depicted the survival outcomes for these groups. The log-rank test was applied to evaluate the statistical significance of the differences observed between the survival curves.

### Enrichment analysis

The FindMarkers function was utilized to identify DEGs with defined thresholds of *P*_adj_ < 0.05 and logFC > 0.25. For Gene Ontology (GO) and Kyoto Encyclopedia of Genes and Genomes (KEGG) analyses, the clusterProfiler package was employed. Additionally, Gene Set Enrichment Analysis (GSEA) and Gene Set Variation Analysis (GSVA) were executed using their dedicated packages.

### Evaluation of immune cell subtype distribution

The study utilized CibersortX and the average expression levels of signature genes for an in-depth analysis of cell subtype infiltration within the TARGET clinical cohort. Using Spearman correlation analysis, the study uncovered the relationships between ZNF451 and diverse cell populations.

### Chemotherapy drug sensitivity analysis

This study delved into the influence of ZNF451 gene expression on chemotherapy efficacy. IC_50_ (median inhibitory concentration) values of 198 drugs, obtained from the Genomics of Drug Sensitivity in Cancer (GDSC) and the Cancer Cell Line Encyclopedia (CCLE), were analyzed using the oncoPredict package in R. Spearman’s correlation was used to assess the association between the drugs’ IC_50_ values and risk scores, thereby pinpointing pertinent drugs. For drugs with a correlation absolute value above 0.2, a comparative analysis of IC_50_ values between high- and low-expression groups was performed. Data visualization was achieved using the ggplot2 package in R.

### Molecular docking investigation

The 3-dimensional (3D) structure of the ZNF451 protein and data on 4 drugs were acquired from the AlphaFold Protein Structure Database [25] and PubChem. Preprocessing steps, including the removal of water molecules and the addition of nonpolar hydrogen atoms, were conducted on the protein and drugs using PyMOL and AutoDock4. Molecular docking was performed with AutoDock4, following the establishment of suitable docking parameters and grid box dimensions. Docking outcomes were visualized via PyMOL, and binding energies below −2.0 kcal/mol were deemed indicative of successful docking.

### Development of cisplatin-resistant osteosarcoma cell lines

Cisplatin-resistant cell lines, termed MG63/R and U-2OS/R, were established from the MG63 and U-2OS cell lines [26]. Initially, MG63 and U-2OS cells were seeded in separate culture dishes, then treated with 1 μM cisplatin, and cultivated for 48 h. After removing cisplatin, cells were cultured in a fresh medium until reaching optimal growth. The cisplatin concentration was incrementally increased, doubling in each of the 6 stages. The successful development of the cisplatin-resistant cell lines was confirmed once the cells demonstrated stable growth in cisplatin concentrations above 32 μM.

### qRT-PCR quantitative analysis

Initially, total RNA was extracted from the samples using the TRIzol (Invitrogen) method, followed by reverse transcription of RNA using the PrimeScript RT reagent kit (TaKaRa, Japan). We prepared reaction mixtures according to the recommendations for the SYBR Green reagent by Takara and performed quantitative real-time polymerase chain reaction (qRT-PCR) analysis on the Roche LightCycler 480 II system. Each sample was set up with 3 replicates.

### siRNA and shRNA design and transfection

We synthesized 3 unique small interfering RNAs (siRNAs) targeting ZNF451 and a singular short hairpin RNA (shRNA) sequence utilizing Genepharma’s siRNA and shRNA design tool, based in Shanghai, China. For shRNA studies, these sequences were integrated into the pLKO.1 vector. The combination of the pLKO.1 shRNA plasmid, along with packaging and envelope protein plasmids, was cotransfected into human embryonic kidney (HEK) 293T cells, facilitating the production of lentiviral particles. Osteosarcoma cells in the logarithmic phase of growth were incubated in 6-well plates until they achieved 70% to 80% confluence, after which transfection was performed using the viral particles, supplemented with Polybrene to enhance efficiency. The cells were then maintained in a standard culture medium for 24 h after a 12-h transfection period before being harvested. The efficacy of transfection was confirmed using qRT-PCR. In the siRNA approach, cells at logarithmic growth were plated in 6-well plates and transfected with Lipofectamine 3000, following the manufacturer’s instructions, when they reached 70% to 80% confluence. After a 6-h transfection period, the medium was switched to a regular culture medium, and the cells were cultured for an additional 24 h prior to collection for further analysis. (The sequences for sh-ZNF451 and si-ZNF451 are detailed in Table S1.)

### 5-Ethynyl-2′-deoxyuridine staining

In accordance with the supplier’s guidelines, the 5-ethynyl-2′-deoxyuridine (EdU) labeling reagent from Apexbio was utilized for cell treatment. Following this, each well-received Click reaction solution containing Cy3 azide was incubated in darkness at room temperature. After phosphate-buffered saline (PBS) wash, Hoechst 33342 solution was applied, and the wells were incubated once more under the same conditions. After completing these steps, the samples were examined under a fluorescence microscope.

### Evaluation of cell viability with CCK-8 assay

Cell viability was evaluated using Sigma-Aldrich’s Cell Counting Kit-8 (CCK-8). In compliance with the manufacturer’s protocol, cells were plated in a 96-well format and permitted to fully adhere prior to specific treatments. Subsequently, the CCK-8 reagent was introduced to each well and incubated under dark conditions at a designated temperature. Cell viability was ascertained by quantifying the absorbance at 450 nm.

### Western blot analysis

Cell lysis was performed using Beyotime’s radioimmunoprecipitation assay (RIPA) buffer, followed by protein concentration measurement with their Bicinchoninic Acid (BCA) Assay kit. Equal amounts of protein were subjected to 10% sodium dodecyl sulfate–polyacrylamide gel electrophoresis (SDS-PAGE) and then transferred onto polyvinylidene difluoride (PVDF) membranes. Membranes were blocked using 5% skim milk and incubated with primary antibodies on a shaker at 4 °C. Subsequently, membranes were incubated with horseradish peroxidase (HRP)-conjugated secondary antibodies from Proteintech at room temperature. After washing, signal detection was conducted using the UVP ChemStudio system (Ultraviolet Products, USA), and data analysis was performed with VisionWorks software (Analytik Jena, Germany). Antibodies used included anti-ZNF451 (1:1,000, Proteintech), anti-BCL2 (1:200, Abcam), anti-BAX (1:1,000, Abcam), anti-E-cadherin (1:5,000, Proteintech), anti-N-cadherin (1:2,000, Proteintech), anti-vimentin (1:1,000, Cell Signaling Technology), anti-SNAI1 (1:1,000, Cell Signaling Technology), and anti-β-tubulin (1:1,000, Santa Cruz Biotechnology).

### Fluorescence immunostaining

In the process of fluorescence immunostaining, paraffin-embedded sections undergo dewaxing and rehydration. This is followed by antigen retrieval in a citrate buffer. To minimize nonspecific binding, the sections are then treated with an antigen-blocking solution at room temperature. Subsequently, they are incubated with a ZNF451 antibody at a low temperature. After washing, the sections are incubated in the dark at room temperature with an Alexa Fluor 555-conjugated immunoglobulin G (IgG) antibody. Finally, the sections are mounted using a medium containing 4′,6-diamidino-2-phenylindole (DAPI) and examined using fluorescence microscopy.

### Detection of apoptotic cells

In this study, cells from both shRNA-ZNF451 and shRNA-NC groups were cultured in 6-well plates and subsequently harvested. After harvest, the cells underwent digestion, centrifugation, and concentration adjustment prior to staining. For the detection of apoptotic cells, we employed Annexin V-FITC (fluorescein isothiocyanate) and propidium iodide staining (BD, UK), followed by analysis through flow cytometry after dark incubation at room temperature. Moreover, as caspase-3 activity is an essential indicator of apoptosis, we utilized the Caspase 3 Activity Assay Kit to process the cells through lysis, centrifugation, and mixing, culminating in a subsequent incubation period. The detection after incubation was conducted using a microplate reader. To corroborate the occurrence of apoptosis, the TUNEL (terminal deoxynucleotidyl transferase-mediated deoxyuridine triphosphate nick end labeling) assay kit was employed for identifying apoptotic cells within tissue samples. These samples, extracted as tumor cryosections from experimental animals, were incubated, stained, washed, mounted, and finally examined under a fluorescence microscope.

### Soft agar colony formation analysis

This study employed a soft agar colony formation assay to assess cellular clonal formation capabilities. Initially, a foundational agar layer was set in 6-well plates. Following the solidification of this layer, a top agar layer, infused with a predetermined quantity of logarithmically growing osteosarcoma cells (subjected to specific transfection protocols), was applied. This layering process facilitated cell culture in a stable temperature- and humidity-controlled incubator until colony formation was evident. To enhance colony visibility, crystal violet staining was utilized, enabling precise enumeration via microscopy. The quantification of these colonies provided a measure of the cells’ clonal formation potential. Rigorous replication in the experimental design ensured the accuracy and reliability of the findings.

### Flow cytometry for cell cycle

Cell cycle detection was performed using the Cell Cycle Staining Kit (MultiSciences, China). Cells were cultured to the logarithmic growth phase, washed with PBS buffer, and digested with 0.25% trypsin-EDTA solution. The cell suspension was collected and centrifuged, the supernatant was discarded, and the cells were resuspended in 1 ml of PBS. After discarding the supernatant, DNA Staining Solution and Permeabilization Solution were added, and the mixture was incubated in the dark for 30 min. Finally, the cells were analyzed using a flow cytometer (CytoFlex SRT, Beckman, USA).

### Isolation of CD8^+^ T cells from osteosarcoma tissue

During osteosarcoma resection surgery, human osteosarcoma tissues were obtained directly from the operating room. After rinsing with PBS (Gibco), the tissues were minced into small pieces and digested in RPMI (Gibco) digestion buffer containing 25 μg/ml Liberase (Roche) and 50 μg/ml DNase (Sigma) at 37 °C for 30 min with shaking. Following mechanical disruption, a single-cell suspension was prepared by passing the sample through a 70-μm filter, and excess red blood cells were removed using a red blood cell lysis buffer. After 2 washes with PBS, the single cells were stained at 4 °C with APC-CY7 fixable viability dye (BD Pharmingen) for 15 min. The samples were then incubated with FcR blocker for 20 min, followed by the addition of cell surface markers. All samples were acquired on a cytoFLEX flow cytometer and analyzed using FlowJo [27]. The following cell surface markers were used: CD3-AF546, CD4-FITC, CD8-PerCP, and PD1-BV510 (Santa Cruz Biotechnology).

### Isolation and culture of peripheral blood CD8^+^ T cells

Peripheral blood lymphocytes were first isolated from healthy donors using density gradient centrifugation with Lymphocyte Separation Medium (Corning). CD8^+^ T cells were then further isolated from the lymphocytes using the EasySep Direct Human CD8^+^ T Cell Isolation Kit (Stemcell Technologies). High CD8-expressing T cells were subsequently validated and sorted using flow cytometry. In all experiments, CD8^+^ T cells were cultured in X-VIVO complete medium supplemented with IL-2 (100 IU/ml, Miltenyi Biotec) and ImmunoCult Human CD3/CD28 T Cell Activator (10 μl/ml, InvivoGen) [27].

### Colony formation assay

Osteosarcoma cells in the logarithmic growth phase were digested and prepared as a single-cell suspension, and 1,000 cells per well were seeded in a 12-well plate. Cells were cultured for 14 d. Then, cells were stained with an appropriate amount of crystal violet staining solution for 30 min.

### Animal experiment

In this study, we utilized six 4-week-old female nonobese diabetic (NOD)–severe combined immunodeficient (SCID) IL2rγnull (NSG) mice, building upon our previous research [28]. These mice were randomly allocated into 2 groups: the sh-ZNF451 group and the sh-NC group, each comprising 3 mice. Each group received inoculations of U2OS/R cells, transfected with either sh-ZNF451 or sh-NC. Tumor sizes were periodically measured using calipers. Upon the tumors reaching a specified average volume, cisplatin (3 mg/kg, twice weekly) was administered intraperitoneally at a predetermined dosage and frequency [29]. The tumor volume was calculated using the following formula: length × width × width/2. Upon conclusion of the experiment, the mice were humanely euthanized, and their tumor tissues were harvested for additional analyses, which included hematoxylin and eosin (H&E) staining and assessments of ZNF451 and TUNEL expression.

### Establishment of patient-derived xenograft model

All human-derived samples used in this study comply with the ethical requirements of the Ministry of Science and Technology of the People’s Republic of China (ethical approval number: [2023]CJ0051). Tumor fragments from osteosarcoma patient biopsies were injected into the subcutaneous tissue of 6-week-old NOD-SCID mice. When the tumor volume reached 100 to 200 mm^3^, the mice were euthanized (P0), and the tumor tissue was minced and injected into another mouse, gradually establishing tumor passages from P1 to P6. Once the P6 generation tumors reached a certain size, tumor-bearing mice were randomly assigned to control and experimental groups [30,31]. The experimental group received a daily oral administration of β-cryptoxanthin at a dose of 10 mg/kg, dissolved in dimethyl sulfoxide (DMSO) [32–34]. β-Cryptoxanthin used in the experiment was purchased from Sigma (catalog number: C6368).

### Micro-CT analysis

Following dissection, femurs and tibias were preserved in 4% paraformaldehyde and subsequently stored at 4 °C in PBS. The specimens were scanned using a high-resolution micro-CT (SkyScan 1276, SkyScan, Aartselaar, Belgium), operating at a resolution of 20.376 μm per pixel, 100-kV voltage, and 200-μA current. The region of interest (ROI) was selected starting 0.45 mm below the distal growth plate and extending proximally for 0.45 mm for trabecular parameter analysis [35], including bone mineral density (BMD), bone surface (BS), bone volume fraction (BV/TV), trabecular thickness (Tb.Th), trabecular number (Tb.N), and trabecular separation (Tb.Sp). Additionally, cortical bone parameters, such as total cross-sectional area inside the periosteal envelope (Tt.Ar), cortical bone area (Ct.Ar), cortical area fraction (Ct.Ar/Tt.Ar), and cortical thickness (Ct.Th), were assessed in a 0.2-mm midshaft section of the femur [36].

## Results

### Single-cell analysis of the osteosarcoma microenvironment

In this study, we analyzed cell classification and gene distribution using data from 2 osteosarcoma databases (GSE152048 and GSE162454), a lymphocyte dataset associated with osteosarcoma (GSE198896), and 2 control bone samples (GSE169396 and GSE217792). To improve data accuracy, cells with elevated mitochondrial gene expression were omitted during the initial processing phase. We employed HVGs and PCA for dimensionality reduction and cell categorization (Fig. S1A and B). Furthermore, the influence of cell cycle-related genes on cell classification was investigated (Fig. S1C), along with an analysis of the correlations between mitochondrial genes, nfeature, erythrocyte-associated genes, and ncount (Fig. S1D to F). Comprehensive visualizations of the nfeature, ncount, and cell cycle-related scores for individual patients are provided (Fig. S1G to J).

**Fig. 1.**
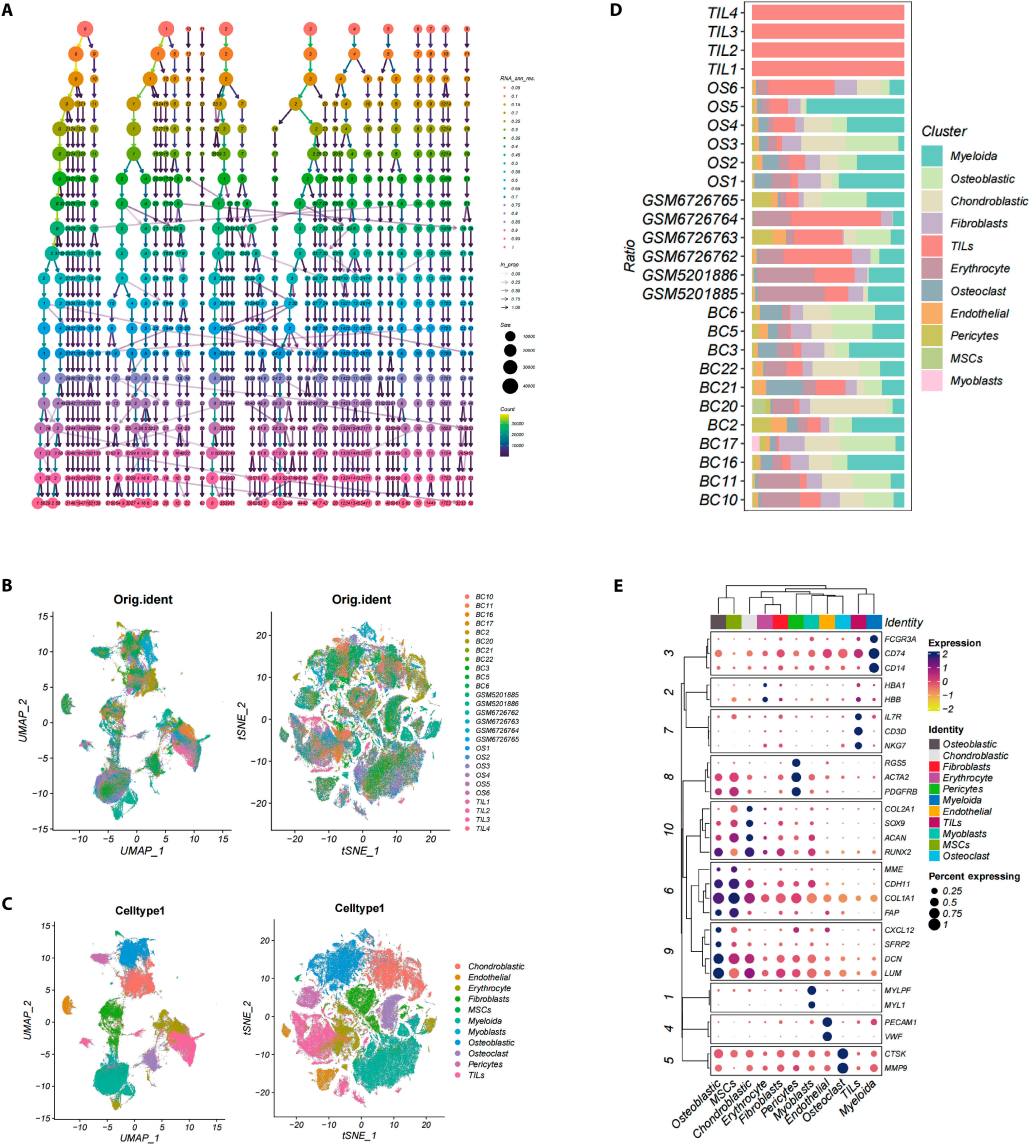
Comprehensive single-cell transcriptomic analysis. (A) Cell population clustering at various resolutions. (B) Spatial distribution of cells from distinct samples illustrated in t-SNE and UMAP visualizations. (C) Classification of predominant cell types as shown in t-SNE and UMAP plots. (D) Relative distribution of cell population clusters within individual samples. (E) Gene expression patterns in diverse cell populations, with dot size denoting the percentage of cells expressing a specific marker gene and color intensity reflecting the mean expression level of that gene.

Following meticulous quality control and batch-effect adjustments, we successfully integrated the data from 191,220 cells. Using the Clustree tool, we depicted the hierarchical structures of cell classifications at different resolutions (Fig. 1A). The t-SNE and UMAP plots, adjusted for batch effects, showed a uniform cell distribution, confirming sample uniformity (Fig. 1B). Using the marker genes cited in a previous study [15], we identified 11 unique cell subpopulations (Fig. 1C). An analysis of cell subgroup proportions across various samples highlighted the intratumoral diversity and shared characteristics of the lesions (Fig. 1D). Furthermore, the genes identified for each cell subgroup were categorized and anchored to their specific highly expressed genes (Fig. 1E).

### Osteoblastic cell heterogeneity in osteosarcoma

**Fig. 2.**
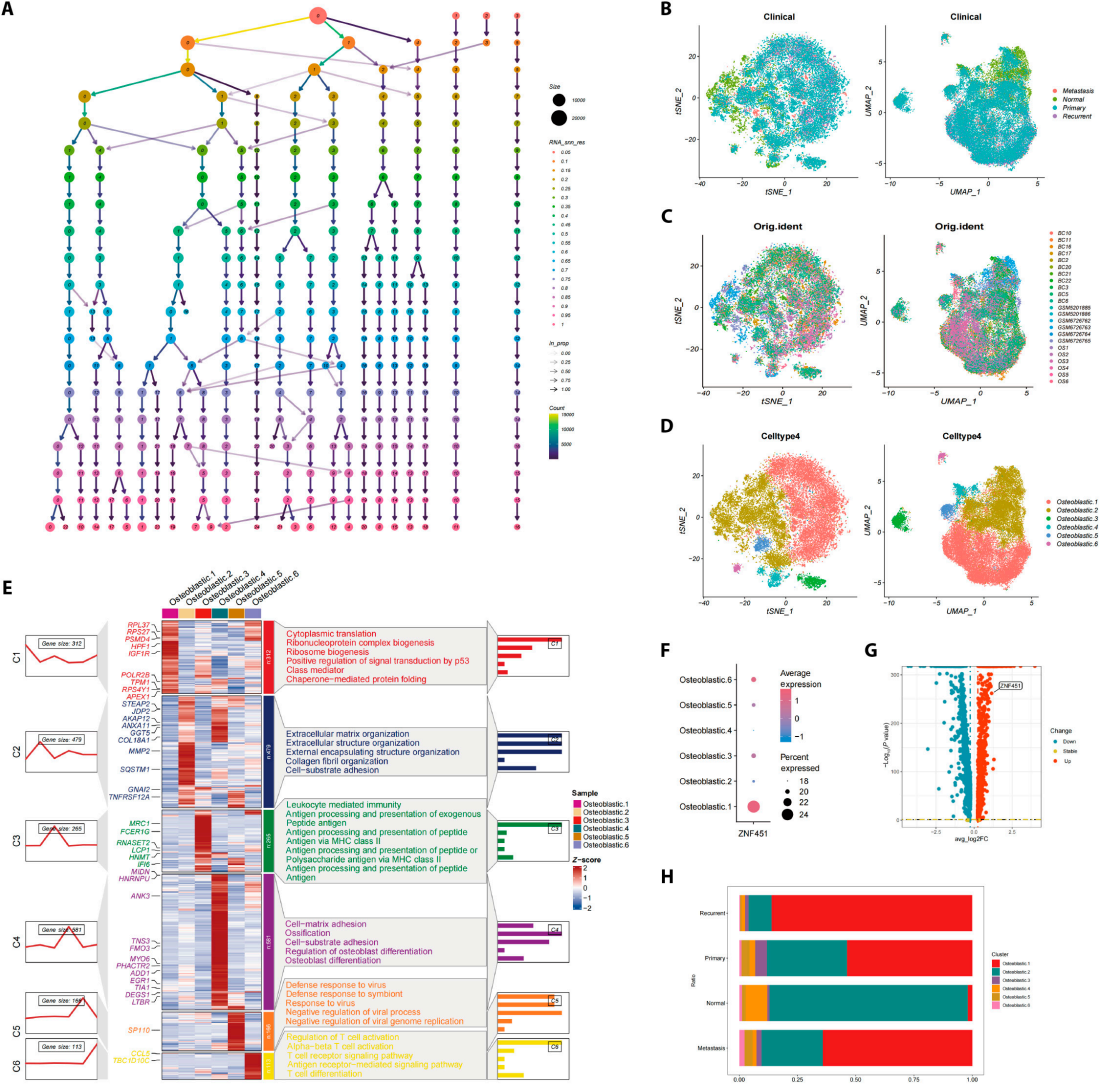
Osteoblast diversity analysis. (A) Cell population clustering across diverse resolutions. (B to D) UMAP and t-SNE visualizations illustrate osteoblast distribution across various clinical types (B), sample sources (C), and principal subgroups (D). (E) Gene expression heatmap for osteoblast subgroups accompanied by GO analysis results. (F) Expression of ZNF451 in 6 subgroups. (G) Volcano plot of DEGs contrasting osteoblasts from primary versus recurrent osteosarcoma, with red highlighting genes overexpressed in recurrent osteosarcoma. (H) Proportional distribution of ZNF451 in osteoblast subgroups across different clinical samples.

This study focused on the heterogeneity of osteoblasts, a key cell subtype of osteosarcoma. We employed the cluster method to generate a multiresolution classification map of osteoblasts (Fig. 2A). Advanced visualization techniques such as t-SNE and UMAP were used to display the clinical information of the identified cells (Fig. 2B), their tissue origins (Fig. 2C), and 6 distinct osteoblastic subgroups (Osteoblastic1 to Osteoblastic6) (Fig. 2D). Each subgroup showed unique gene expression profiles and pathway enrichment patterns (Fig. 2E), suggesting diverse functional roles in vivo. GO enrichment analysis revealed several distinct characteristics of these subgroups. Osteoblastic1 osteosarcoma cells exhibited high levels of protein synthesis and ribosome biogenesis activity, as well as significant RNA processing and translation regulation capabilities, along with p53-mediated signaling regulation and molecular chaperone-mediated protein folding functions. These characteristics may confer high proliferative potential, anti-apoptotic ability, and adaptability to drug treatment. Osteoblastic2 cells displayed functions related to extracellular matrix remodeling, extracellular structure organization, collagen fiber formation, and cell-matrix adhesion, suggesting a potential role in tumor invasion, metastasis, and microenvironment regulation. Osteoblastic3 osteosarcoma cells are active in antigen processing and presentation, and leukocyte-mediated immune functions, indicating a potential role in modulating tumor immune responses, which may influence immune evasion mechanisms in the tumor. Osteoblastic4 osteosarcoma cells were enriched in cell-matrix adhesion, osteoblast differentiation, and ossification, implying a key role in bone tissue formation and tumor cell invasion. Osteoblastic5 osteosarcoma cells exhibit significant functions in antiviral responses, symbiont defense, and inhibition of viral replication, Osteoblastic6 cells showed significant activity in regulating T cell activation and differentiation, potentially affecting the immune microenvironment, tumor progression, and response to therapy (Fig. 2E). Notably, *ZNF451* is highly expressed in Osteoblastic1 and Osteoblastic6, and these subgroups are closely associated with high proliferative potential, anti-apoptotic ability, drug treatment adaptability, and T cell regulatory functions (Fig. 2F). Notably, osteoblastic cells in recurrent osteosarcoma presented substantial variance in gene expression compared to those in primary osteosarcoma (Fig. 2G). Additionally, the proportion of each osteoblast subpopulation varied significantly across different disease types, with the *ZNF451*-high Osteoblastic1 subpopulation being scarce in normal tissues but significantly increased in osteosarcoma tissues. Notably, the proportion was much higher in recurrent osteosarcoma compared than in primary osteosarcoma. These findings support the crucial role of *ZNF451* in the initiation, progression, and development of osteosarcoma (Fig. 2H).

### Myeloid cell dynamics in osteosarcoma’s immune microenvironment

Within the immune microenvironment of osteosarcoma, myeloid cells exhibit a dichotomous role: They contribute to both tumor growth and invasion, but also significantly influence immune response modulation and treatment outcomes. Through meticulous multiresolution analysis, we identified the ideal resolution for effectively differentiating various cell subpopulations (Fig. S2A). Using t-SNE technology, we mapped the spatial distribution of myeloid cells and observed no direct correlation with individual samples, which validated the removal of potential batch variances. We identified 6 distinct cell subpopulations (Fig. S2B) and used a dot plot to thoroughly examine the signature genes of these cells (Fig. S2C). GO enrichment analysis revealed the unique biological functions inherent to each myeloid cell type (Fig. S2D). Furthermore, a comparative analysis revealed notable transcriptional discrepancies between myeloid cells in primary and recurrent osteosarcoma (Fig. S2E), as well as significant variability distribution of myeloid cell distribution across osteosarcoma samples (Fig. S2E).

### Analysis of tumor-infiltrating lymphocyte subgroups in osteosarcoma

Tumor-infiltrating lymphocytes (TILs) play a central role in the tumor microenvironment, significantly influencing immune regulation and the efficacy of therapeutic interventions. Extensive research have emphasized the critical role of TILs in the success of immunotherapy. Utilizing optimal resolution (Fig. S3A), our UMAP-based analysis clearly identified TIL marker genes, which are instrumental in differentiating various cell clusters (Fig. S3B to E and G). Additionally, a comparative study revealed pronounced transcriptional variance between TILs in recurrent and primary osteosarcoma tissues (Fig. S3F).

### Investigating intercellular communication in the tumor microenvironment

**Fig. 3.**
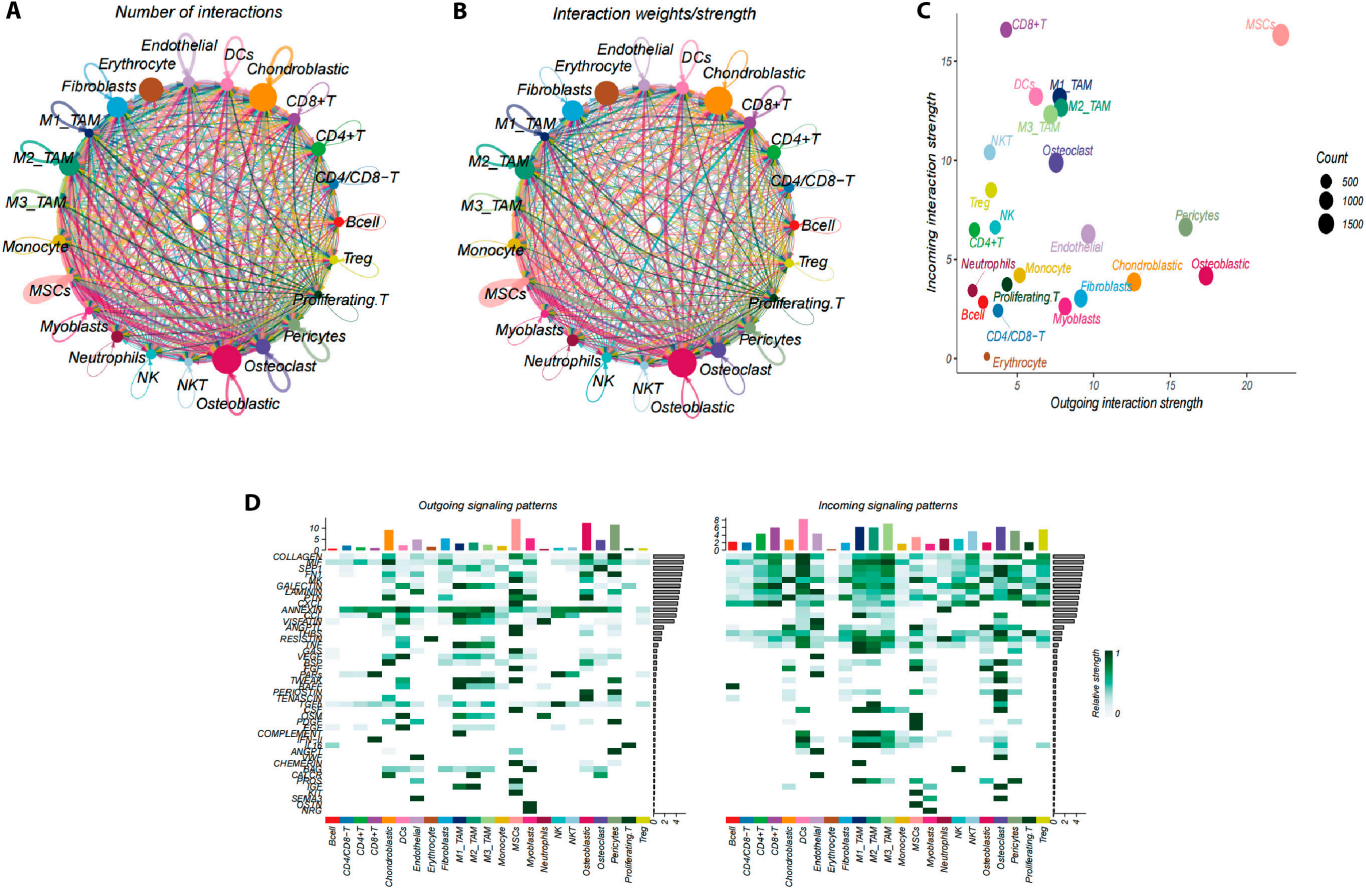
Comprehensive analysis of intercell subpopulation communication networks. (A and B) Illustration of the frequency and magnitude of interactions among various cell subpopulations. (C) Visual representation of key signal transmitters (sources) and recipients (targets). (D) Identification of major trends in signal transmittal and reception.

Our study revealed extensive intercellular communication among various cell subgroups (Fig. 3A and B and Figs. S4 and S5). Within this network, osteoblastic cells were identified as the primary signal transmitters, whereas CD8^+^ T cells predominantly acted as signal receivers. Notably, mesenchymal stem cells (MSCs) uniquely acted as both significant senders and receivers of signals (Fig. 3C). Moreover, osteoblasts demonstrated the ability to interact with diverse cell subgroups via multiple signaling pathways (Fig. S6). To further elucidate these interactions, we identified key communication signals, including GALECTIN and MIF (Fig. 3D).

To explore intercellular interaction patterns more deeply, we integrated the CellChat approach with advanced pattern recognition techniques. Using the Silhouette method, we precisely identified and categorized various interaction patterns (Fig. S7A, B, E, and F). Sankey diagrams effectively demonstrated the connections among different cell subgroups, their communication networks, and associated signaling pathways (Fig. S7C and G). In addition, we conducted a comprehensive analysis of the evolving trends in key signaling pathways using pattern analysis techniques (Fig. S7D and H). Notably, cells including CD8^+^ T, CD4^+^ T, and regulatory T cells were identified to play pivotal roles in this communication network.

### Differential intercellular communication in recurrent versus primary osteosarcoma

**Fig. 4.**
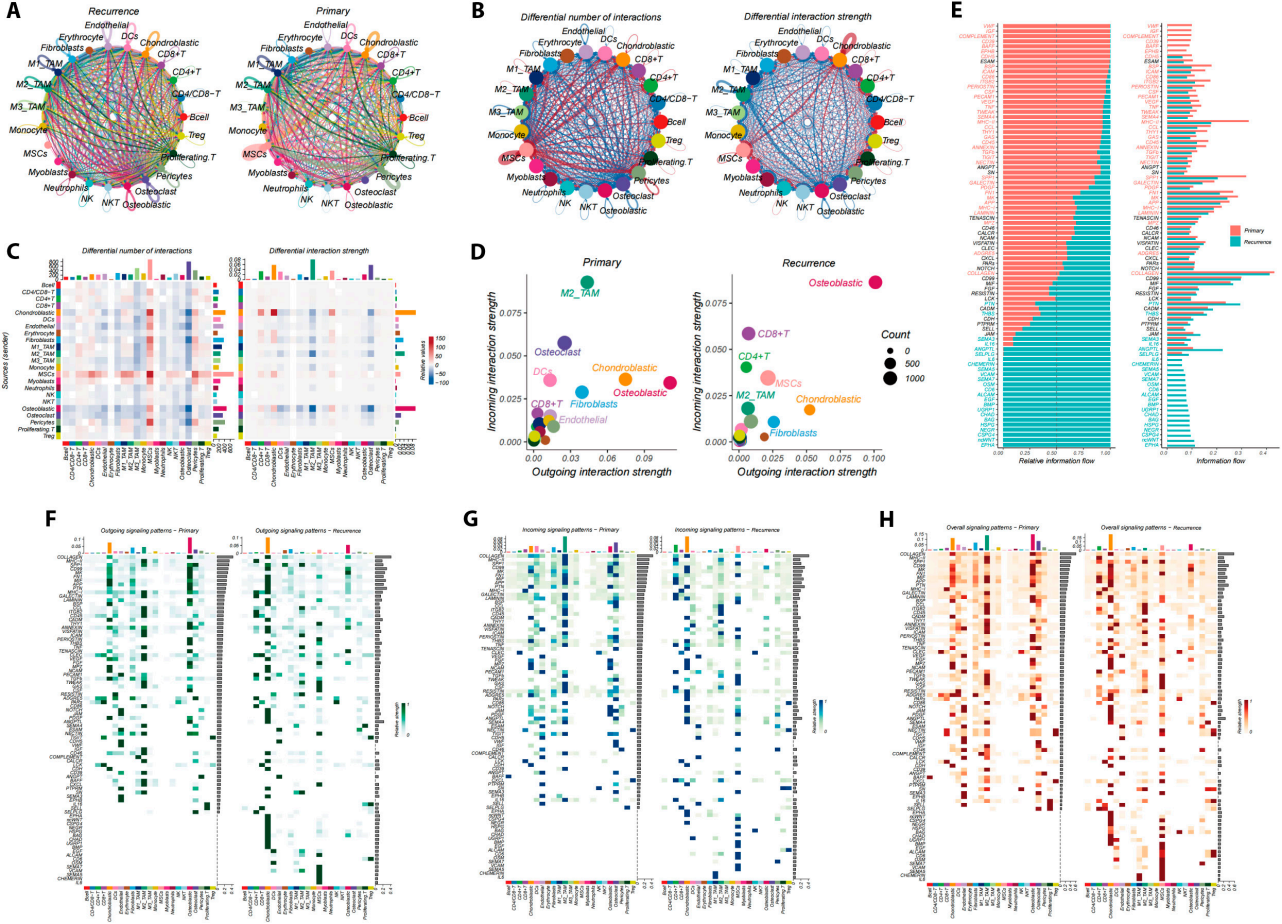
Comparative analysis of intercellular communication in recurrent versus primary osteosarcomas. (A) Communication networks within recurrent and primary osteosarcoma tissues. (B) Variations in the frequency and strength of intercellular interactions in recurrent osteosarcomas, with red signifying enhancement and blue indicating reduction. (C) Heatmap detailing the disparities in interaction frequency and intensity across recurrent and primary osteosarcoma tissues. (D) Comparative evaluation of principal signal emitters and receivers in both recurrent and primary osteosarcomas. (E) Expression of key signaling molecules and pathways. (F) Outgoing signal analysis across different cell subgroups. (G) Incoming signal analysis across different cell subgroups. (H) Comprehensive signal transmission assessment among various cell subgroups.

Our study further investigated the role of intercellular communication in osteosarcoma progression by comparing the communication networks in recurrent and primary osteosarcoma tissues. Although both forms exhibited intricate communication networks (Fig. 4A), significant differences were observed in the frequency and intensity of interactions between recurrent and primary forms (Fig. 4B). Analysis of communication among cell subgroups identified osteoblastic cells as the primary signal transmitters (Fig. 4C and D). Further investigation revealed elevated expression of key signaling molecules and pathways, such as epidermal growth factor (EGF) and interleukin-6 (IL-6), in recurrent osteosarcomas compared to their primary counterparts (Fig. 4E). These up-regulated signals showed significant associations with SUMOylation modifications, implying a potential role for SUMOylation in osteosarcoma recurrence [37–40]. Comparative analyses of signaling pathways, ligands, and receptor interactions further highlighted significant differences in signal dynamics and overall communication patterns relative to the control bone tissue (Fig. 4F to H and Fig. S8).

### Role of *ZNF451* in osteosarcoma

**Fig. 5.**
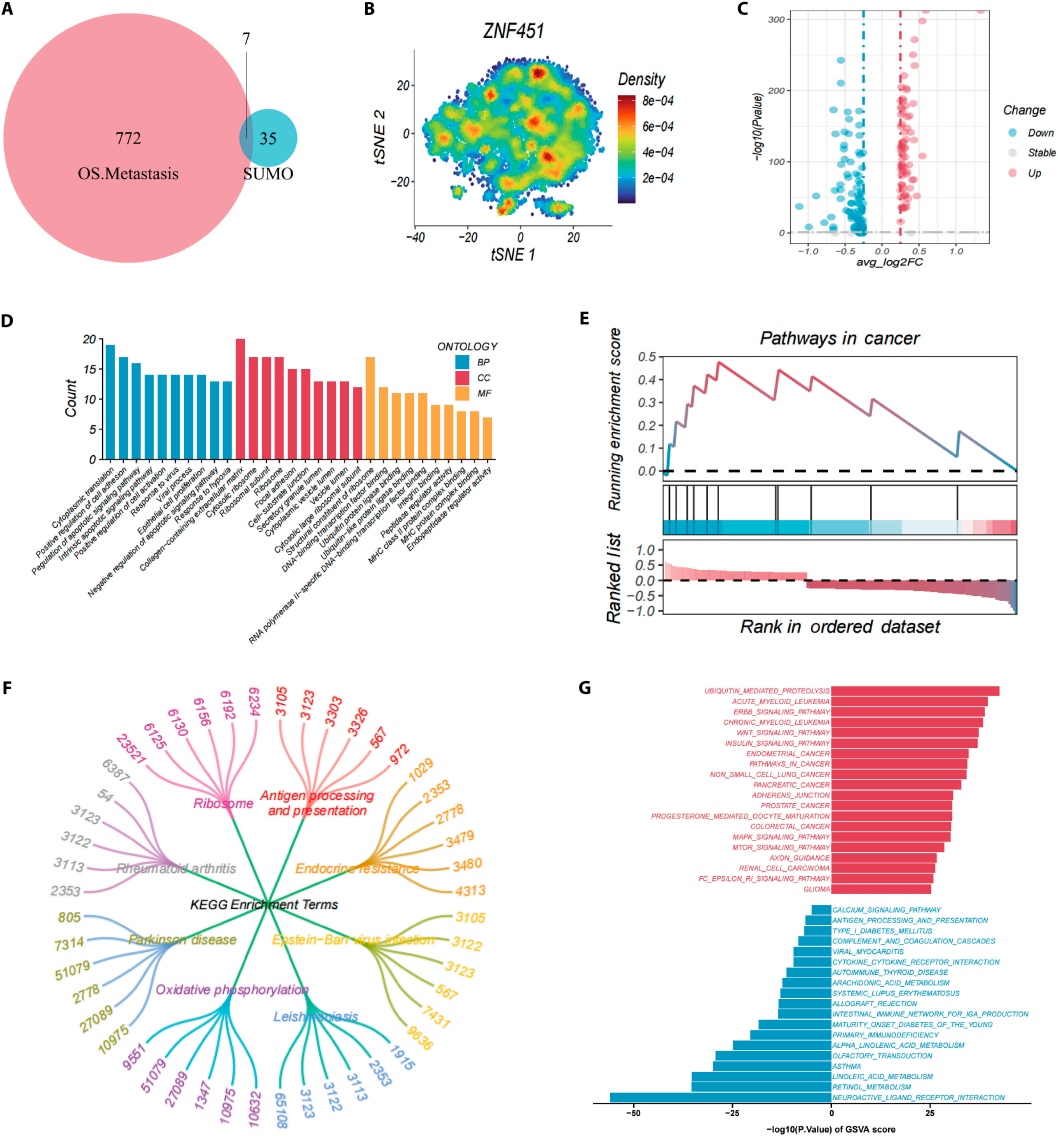
Functional role of ZNF451 in osteosarcoma. (A) Venn diagram showing the overlap between SUMOylation-related genes and genes highly expressed in osteosarcoma. (B) t-SNE plot illustrating expression variations of ZNF451 in osteoblasts. (C) Volcano plot depicting ZNF451 expression disparities. (D) GO analysis of genes with differential expression. (E) GSEA of DEGs. (F) Analysis of DEGs using the KEGG database. (G) GSVA of genes exhibiting differential expression.

To explore the connection between SUMOylation and osteoblastic osteosarcoma, we examined 42 genes linked to SUMOylation [41,42]. By comparing these genes with 772 genes uniquely overexpressed in recurrent osteoblastic osteosarcoma, we identified 7 key genes, termed OS-SUMOs (Fig. 5A). The prognostic significance of OS-SUMOs was using the GSE21257 osteosarcoma dataset, where *ZNF451*, a SUMOylation-related gene, emerged as a key predictor of patient survival (Fig. S9). The expression dynamics of *ZNF451* in osteosarcoma cells were extensively studied (Fig. 5B), stratifying cells into high and low *ZNF451* expression categories based on their median expression levels, and disparities in gene expression are visually represented in a volcano plot (Fig. 5C).

GO enrichment analysis suggested that *ZNF451* is involved in various cellular functions (Fig. 5D), impacting processes such as intracellular protein synthesis, enhancement of epithelial–mesenchymal transition (EMT), apoptotic signaling pathways (both promotion and inhibition of apoptosis), cellular activation, viral response, epithelial cell proliferation, and adaptation to hypoxia. *ZNF451* was linked to components of the collagen-rich extracellular matrix, ribosomes and their subunits, focal adhesion sites, cell-matrix connections, and intracellular vesicle formation. In terms of molecular functions, *ZNF451* may contribute to ribosomal structure, interact with DNA-binding transcription factors, bind to ubiquitin–protein ligases and integrins, and regulate several enzymatic processes. These findings suggest that *ZNF451* plays a crucial role in osteosarcoma by influencing cellular proliferation, differentiation, EMT, and migration. Specifically, *ZNF451* may enhance cell proliferation via intracellular protein synthesis, and affect tumor cell mobility and invasiveness by regulating EMT. Its effect on apoptotic signaling pathways may be crucial for determining cell fate, impacting osteosarcoma development and progression. Additionally, *ZNF451*’s response to viral attacks and low-oxygen environments may also reflect its adaptability to the tumor microenvironment. Collectively, *ZNF451* is hypothesized to be a critical regulator of the biological behavior of osteosarcoma, influencing tumor growth, cellular movement, invasion, and environmental stress responses.

The results from GSEA on “Pathways in Cancer” revealed that *ZNF451* may play a substantial role in numerous cancer-related pathways (Fig. 5E). These findings suggest a strong association between *ZNF451* expression and a range of cancer-related biological pathways that could significantly affect tumor development, progression, and response to treatment. *ZNF451* likely participates in critical regulatory processes, including cell proliferation, apoptosis, cell cycle control, signal transduction, cell migration, and invasion, as well as in the dynamics of the tumor microenvironment.

The KEGG enrichment analysis (Fig. 5F) indicated that *ZNF451* potentially affects ribosomal function, which may subsequently influence protein synthesis and tumor cell growth. *ZNF451*’s involvement in antigen processing and presentation suggests a role in tumor immune evasion, altering the immune landscape. Its link to endocrine resistance pathways hints at a potential role in osteosarcoma’s hormonal response and drug resistance strategies. Furthermore, its role in oxidative phosphorylation and the Leishmania pathway suggests that it influences energy metabolism. Collectively, these pathways suggest the critical involvement of *ZNF451* in the evolution, progression, and therapeutic response in osteosarcoma.

GSVA (Fig. 5G) revealed a potential positive correlation between *ZNF451* expression and several key biological pathways, including ubiquitin-mediated protein degradation and various cancer-associated pathways such as acute and chronic myeloid leukemia, endocrine cancers, non-small cell lung cancer, pancreatic cancer, prostate cancer, colorectal cancer, and glioma. Pathways such as WNT, insulin, mitogen-activated protein kinase (MAPK), and mechanistic target of rapamycin (mTOR) were also implicated, suggesting involvement in cellular proliferation, differentiation, metabolism, and signal transduction. Conversely, *ZNF451* expression negatively correlated with various immune and metabolic pathways, including calcium signaling, antigen processing and presentation, and cytokine–cytokine receptor interactions, hinting at a regulatory role in immune responses and metabolic functions.

The integration of GO, KEGG, GSVA, and GSEA suggests that *ZNF451* plays a complex and multifaceted role in osteosarcoma, influencing ribosomal function, protein synthesis, cell proliferation, apoptosis, EMT, and migration. Moreover, *ZNF451* is implicated in a variety of cancer-related pathways such as ubiquitin-mediated protein degradation and the WNT and ERBB signaling pathways, highlighting its significance in cell growth, differentiation, and signal transduction, as well as immune evasion mechanisms in the tumor microenvironment.

### *ZNF451*’s role in osteosarcoma’s immune microenvironment

**Fig. 6.**
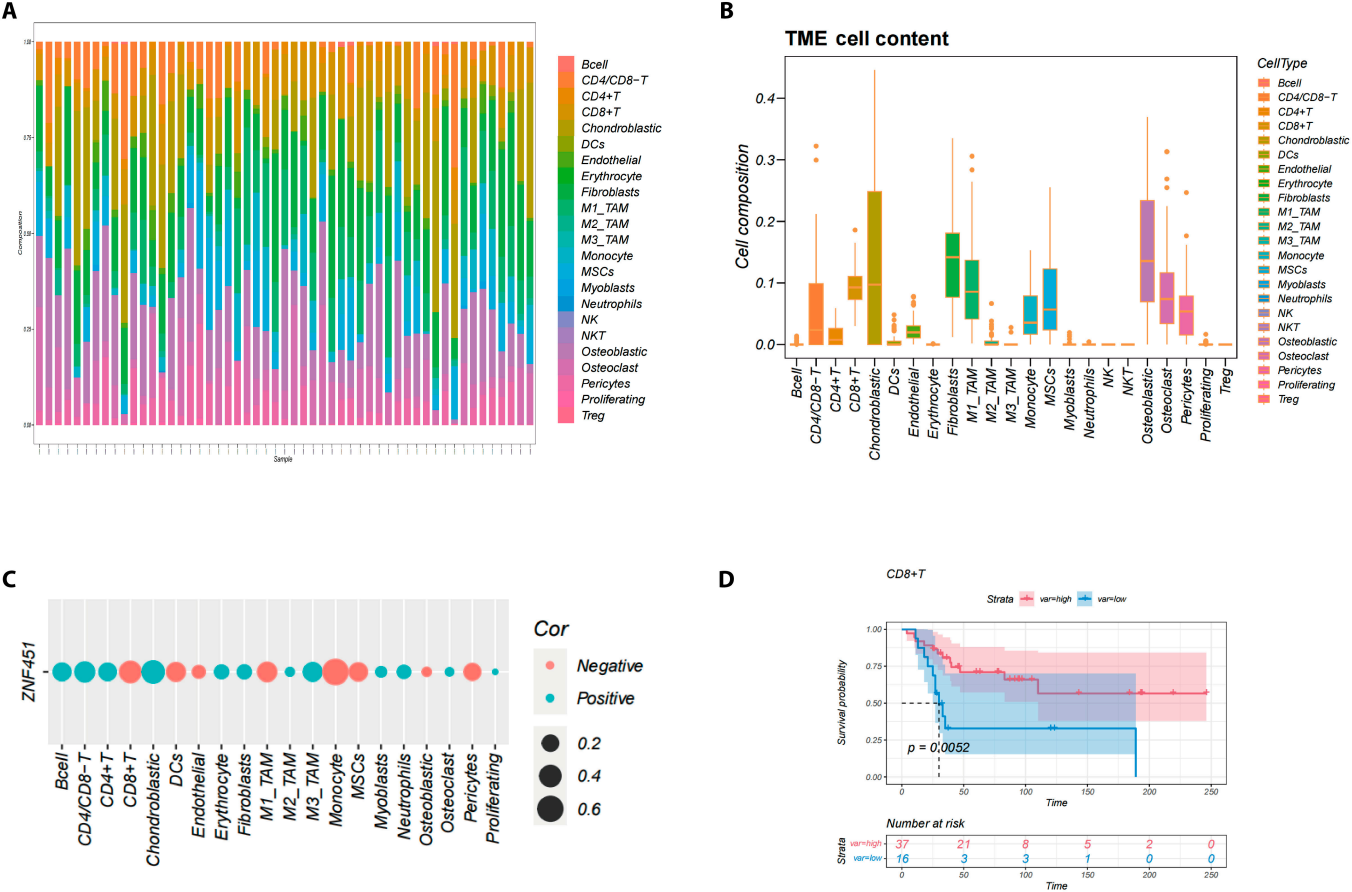
Investigating ZNF451’s impact on the tumor immune microenvironment (TIME). (A) Depiction of immune cell infiltration in diverse samples. (B) Proportional distribution of cell types across all specimens. (C) Correlation assessment between ZNF451 and tumor microenvironment cells. (D) ZNF451-associated survival analysis in CD8^+^ T cells.

To assess *ZNF451*’s impact on the osteosarcoma immune microenvironment, we employed CibersortX, a technique that leverages marker-specific scRNA data, to analyze immune cell composition in TARGET-OS. The analysis, (Fig. 6A) revealed substantial heterogeneity in immune cell infiltration within osteosarcoma, with osteoblasts and chondroblasts being the primary constituents (Fig. 6B). *ZNF451* was significantly associated with most of the infiltrating cells, suggesting its potential influence on osteosarcoma treatment outcomes by modulating the immune microenvironment, as shown in Fig. 6C. Consistent with previous studies, patients with high CD8^+^ T cell expression demonstrated a markedly improved prognosis (Fig. 6D).

### Elevated *ZNF451* expression and its potential role in augmenting chemotherapy resistance in osteosarcoma

**Fig. 7.**
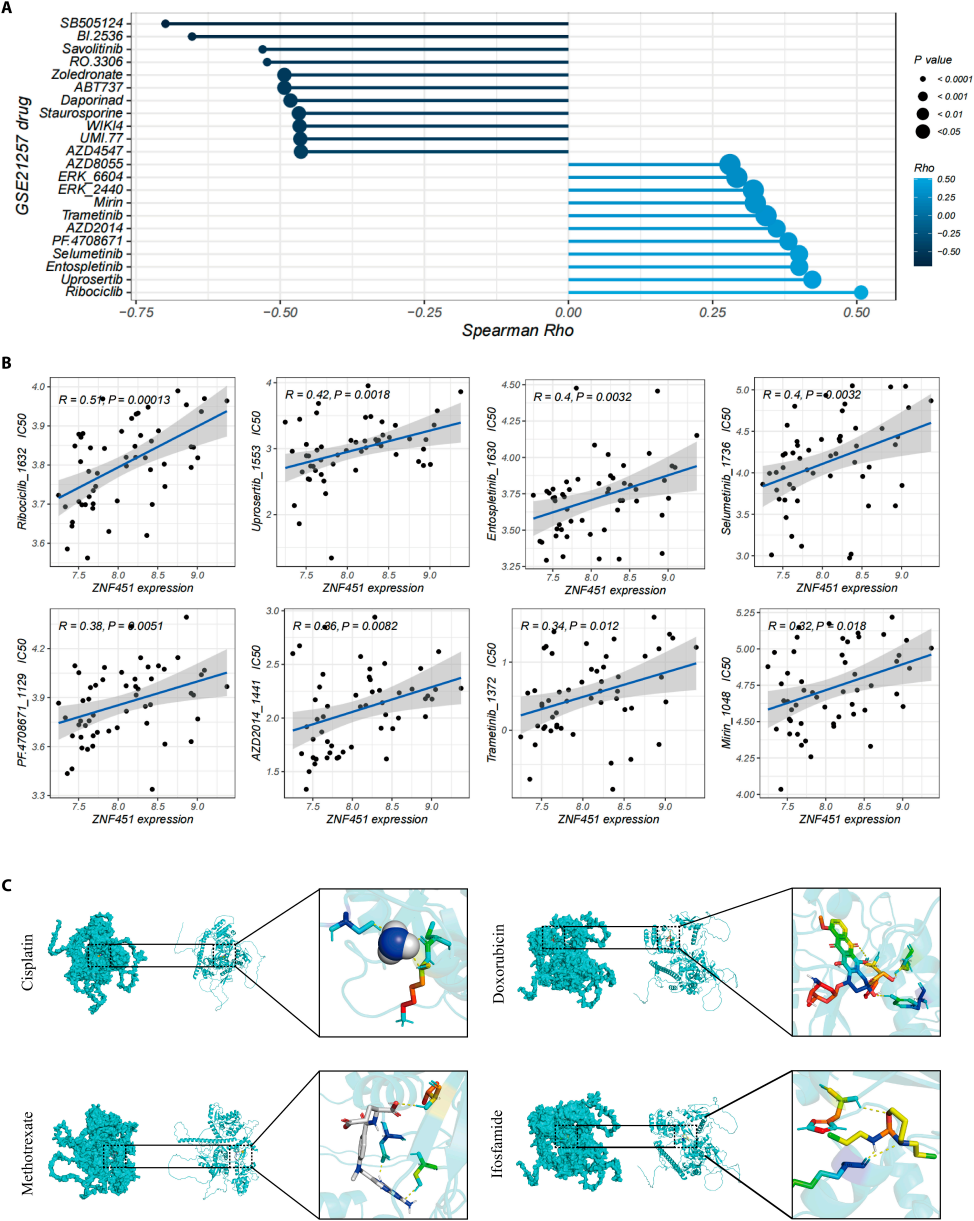
Correlation between elevated ZNF451 expression and enhanced drug resistance in osteosarcoma. (A and B) Examination of ZNF451 expression and its association with the half-maximal inhibitory concentration (IC_50_) of several drugs using the GSE21257 database. (C) Molecular docking studies of ZNF451 with prevalent first-line osteosarcoma medications.

Using the OncoPredict tool and the GSE21257 database, we assessed drug sensitivity in relation to *ZNF451* expression, including IC_50_ values of several key chemotherapy agents, with correlations calculated via Spearman’s method (Fig. 7A and B). These results suggested a potential link between increased *ZNF451* expression and chemotherapy resistance in osteosarcoma. Molecular docking studies revealed a notable affinity between *ZNF451* and primary osteosarcoma treatment drugs (Fig. 7C).

### Role of *ZNF451* in CD8^+^ T cell exhaustion dynamics

**Fig. 8.**
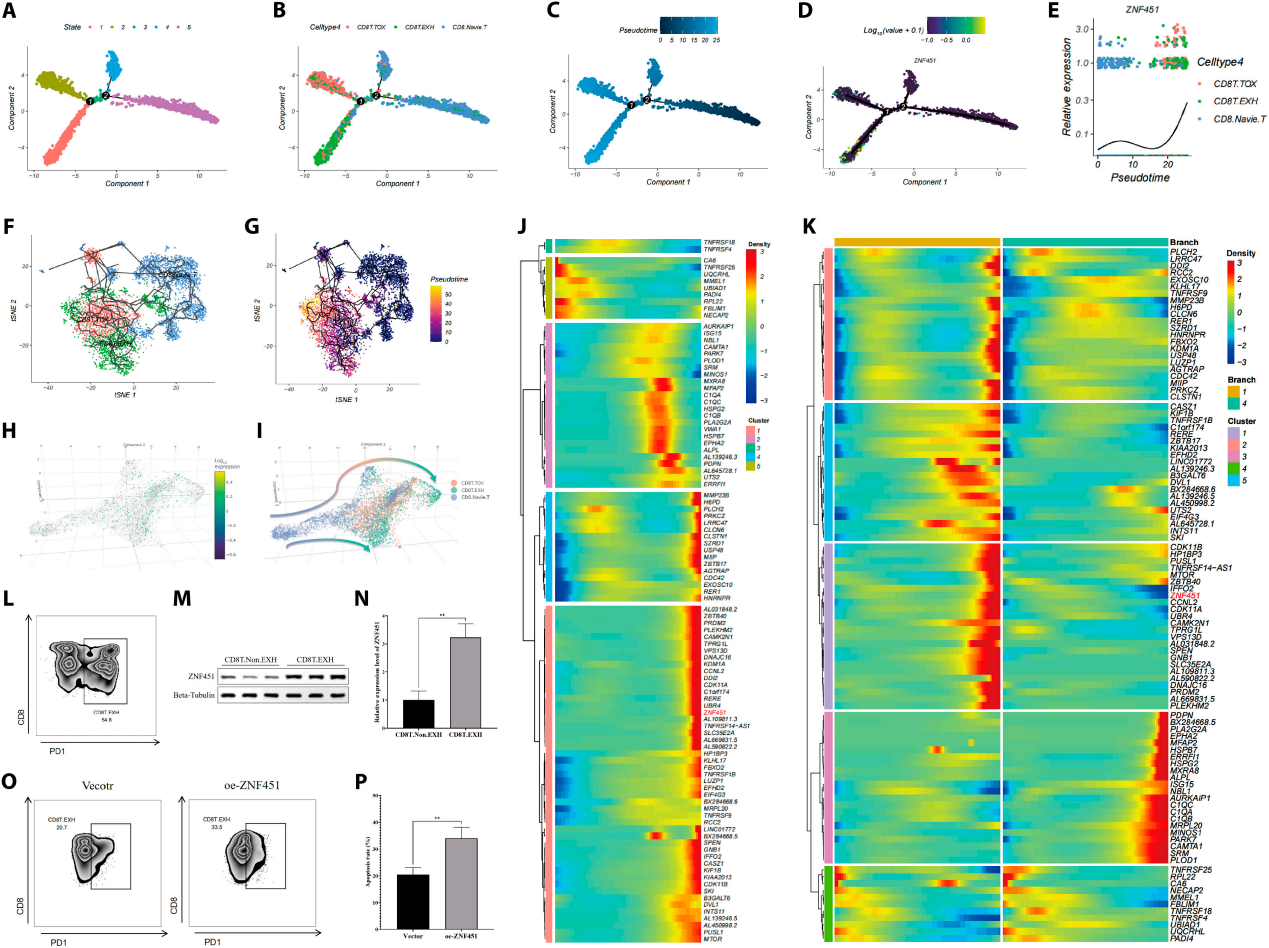
ZNF451-induced CD8^+^ T cell exhaustion. (A to E) Monocle 2 trajectory analyses: CD8^+^ T cell clustering (A), cell type identification (B), pseudotime trajectories (C), ZNF451 expression levels (D), and ZNF451 expression dynamics over pseudotime (E). (F and G) Monocle 3 trajectories depicting CD8^+^ T cell subgroup differentiation (F) and pseudotime progression (G). (H and I) 3D Monocle 3 visualizations of ZNF451 expression fluctuations (H) and cell type dynamics (I). (J) Heatmap of hierarchical clustering displaying developmental time and cell subgroup-associated marker genes. (K) Comparative heatmap clustering illustrating marker genes for different branches, indicating CD8T.EXH differentiation in branch 1 and CD8T.TOX differentiation in branch 4. (L) Flow cytometry sorting of CD8T.EXH and CD8T.Non.EXH cells from osteosarcoma TILs. (M and N) Western blot analysis of ZNF451 expression levels in CD8T.EXH and CD8T.Non.EXH cells. (O and P) Flow cytometry analysis of the proportion of CD8T.EXH cells.

CD8^+^ T cells were isolated and categorized into 3 primary subtypes using precise resolution criteria: CD8.Navie.T (naïve CD8^+^ T cells), CD8T.TOX (cytotoxic CD8^+^ T cells), and CD8T.EXH (exhausted CD8^+^ T cells), as illustrated in Fig. S10A to C. Each subtype displayed unique gene expression profiles (Fig. S10D and E). Notably, *ZNF451* was predominantly expressed in the CD8T.EXH cells (Fig. S10D). To analyze the spatiotemporal development of CD8^+^ T cells, we applied the Monocle 2 and Monocle 3 algorithms for a comprehensive pseudo-temporal analysis. Monocle 2 facilitated the organization of cells and visualization of their trajectory, as shown in Fig. 8A (cell clusters), Fig. 8B (cell classification), Fig. 8C (pseudo-temporal analysis results), Fig. 8D (*ZNF451* expression levels), and Fig. 8E (dynamic changes in *ZNF451* expression over time). *ZNF451* expression initially increased, decreased, and then increased again during the pseudotemporal sequence (Fig. 8E and J). These single-cell trajectories elucidated the cell differentiation pathways based on varying gene expression profiles. Importantly, as depicted in Fig. 8B, at the critical branching point 1, naive CD8^+^ T cells (CD8.Navie.T) differentiated toward both cytotoxic CD8^+^ T cells (CD8T.TOX) and exhausted CD8^+^ T cells (CD8T.EXH), with the latter pathway showing a significant increase in *ZNF451* expression (Fig. 8E and K). Moreover, the expression of *ZNF451* during CD8^+^ T cell differentiation exhibited complex fluctuations, correlating with the incremental stages of CD8T.EXH cell proliferation (Fig. 8I and L). Monocle 3 analysis provided a more visual representation of the temporal and spatial progression from CD8.Navie.T to CD8T.TOX and CD8T.EXH, as well as the transition from CD8T.TOX to CD8T.EXH (Fig. 8F to I). Exhausted CD8^+^ T cells typically exhibit elevated PD1 expression; therefore, many previous studies have used this gene as a marker for identifying CD8^+^ T cells [43–45]. To validate the bioinformatic results, we used PD1 as a marker gene for exhausted T cells and sorted CD8T.EXH and CD8T.Non.EXH cells from patients with osteosarcoma samples using flow cytometry (Fig. 8L and Fig. S10F). Subsequently, we detected *ZNF451* expression via Western blotting and found that CD8T.EXH cells exhibited significantly higher expression compared to CD8T.Non.EXH cells (Fig. 8M and N). To further explore whether *ZNF451* induces CD8^+^ T cell exhaustion, we isolated CD8^+^ T cells from healthy individuals (Fig. S10G). Following *ZNF451* overexpression, flow cytometry was used to assess the proportion of exhausted CD8^+^ T cells (Fig. S10H). The results showed that *ZNF451* overexpression increased PD1 expression in CD8^+^ T cells, thereby inducing CD8^+^ T cell exhaustion (Fig. 8O and P). These findings indicated that *ZNF451* regulates PD1 expression in CD8^+^ T cells, thereby modulating their activation state.

### Evaluating *ZNF451* expression across diverse osteosarcoma cell lines

**Fig. 9.**
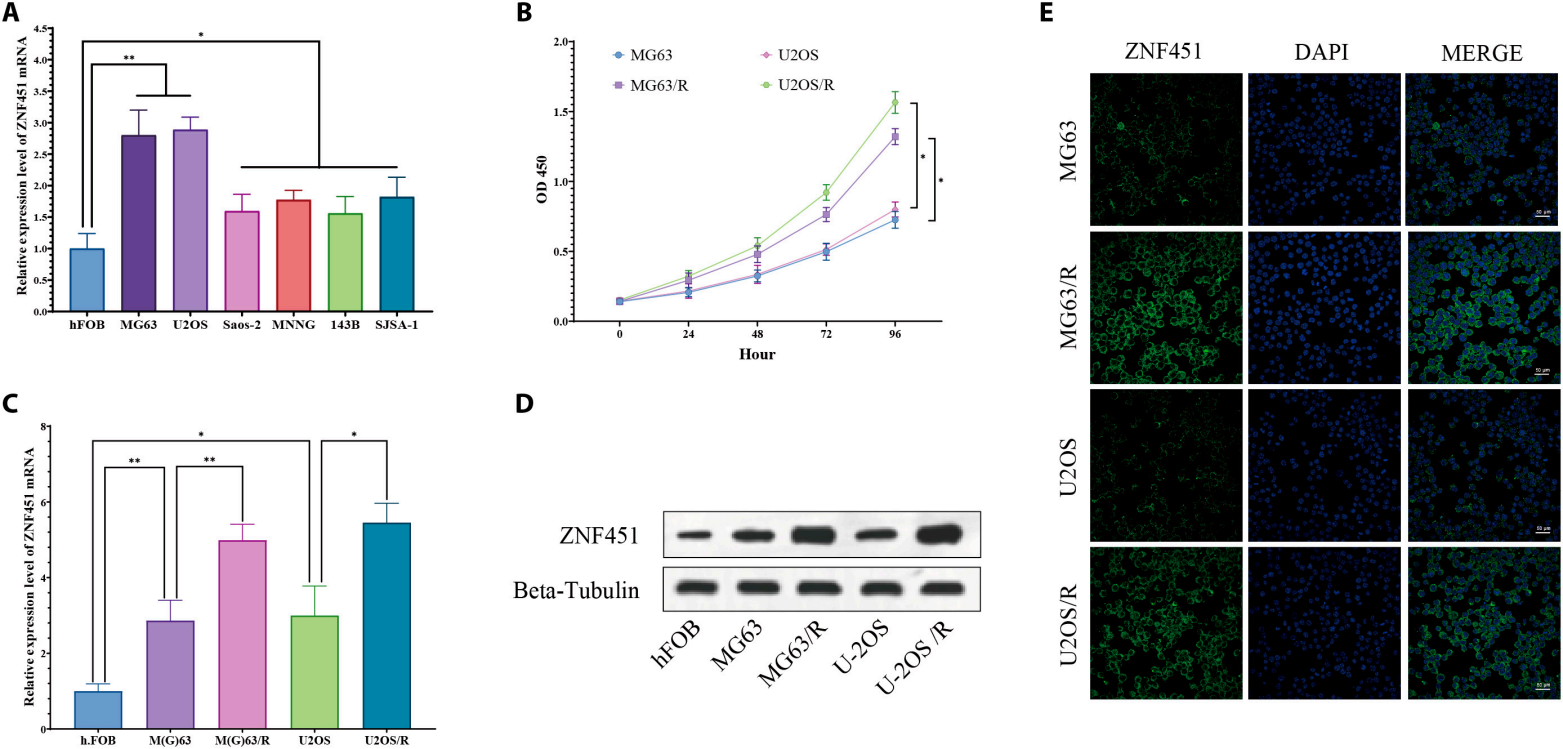
ZNF451 expression in osteosarcoma cell lines. (A) qRT-PCR analysis comparing ZNF451 expression between osteosarcoma cell lines and normal hFOB cells (*n* = 3). (B) Growth rates of U2OS, MG63, MG63/R, and U2OS/R cell lines were quantified via CCK-8 assay (*n* = 5). (C and D) qRT-PCR and Western blot assays to measure ZNF451 expression in U2OS, MG63, MG63/R, and U2OS/R cell lines (*n* = 3). (E) Confocal microscopy to visualize ZNF451 localization in U2OS, MG63, MG63/R, and U2OS/R cells. Scale bar, 50 μm. Statistical significance indicated as *P < 0.05, **P < 0.01, and ***P < 0.001.

In our study of *ZNF451*’s role in osteosarcoma, a variety of osteosarcoma cell lines (U2OS, Saos-2, MG63, 143 B, MNNG, and SJSA-1) and normal osteoblasts (hFOB) were selected for mRNA expression analysis. qRT-PCR consistently showed elevated *ZNF451* mRNA levels in all osteosarcoma cell lines compared to hFOB cells, with notably higher levels in U2OS and MG63 cells (*n* = 3; Fig. 9A).

We developed 2 cisplatin-resistant osteosarcoma cell variants, MG63/R and U2OS/R (*n* = 3; Fig. S11A), using a gradient adaptation approach. Initially, the IC_50_ values for MG63 and U2OS cells against cisplatin were 8.45 μM and 9.46 μM, respectively. After resistance, these values increased to 52.28 μM for MG63/R and 62.61 μM for U2OS/R. Remarkably, the proliferation rates of these drug-resistant cells significantly exceeded those of the original U2OS and MG63 cells at 72 and 96 h (*n* = 5; Fig. 9B). qRT-PCR and Western blot analyses showed that *ZNF451* expression was markedly lower in MG63 and U2OS cells than in the resistant variants MG63/R and U2OS/R (*n* = 3; Fig. 9C and D and Fig. S11B). Immunofluorescence staining showed that *ZNF451* was predominantly localized in the cytoplasm of osteosarcoma cells (Fig. 9E).

### Impact of *ZNF451* on osteosarcoma cells in vitro

**Fig. 10.**
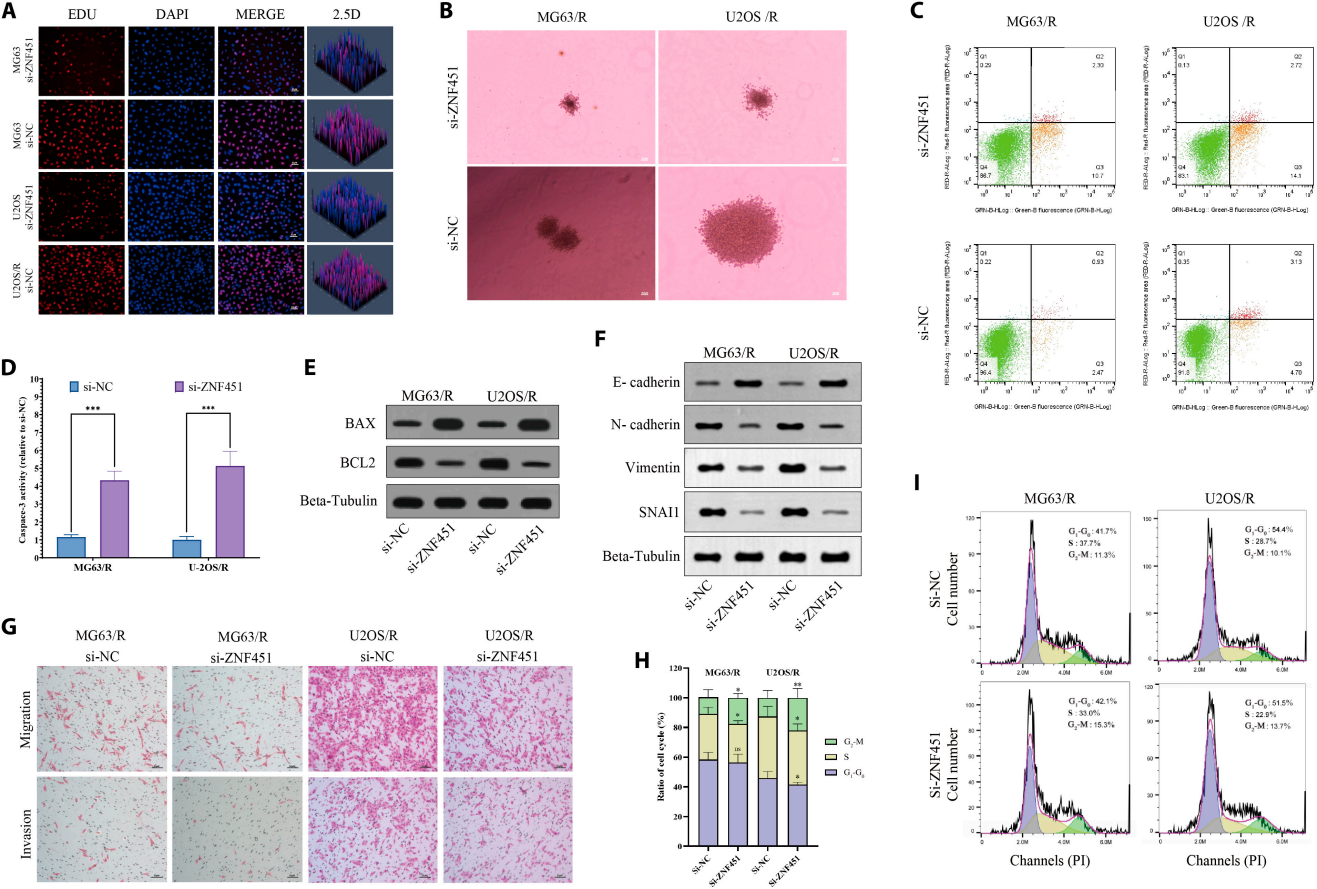
Investigating the impact of ZNF451 on in vitro osteosarcoma drug-resistant cell lines. (A) EdU staining method to assess proliferation in both sensitive and resistant osteosarcoma cell lines (*n* = 3). Scale bar, 20 μm. (B) Soft agar colony formation assay to evaluate the effects of ZNF451 knockdown in drug-resistant cells (*n* = 3). Scale bar, 200 μm. (C) Flow cytometry for determining apoptosis rates in osteosarcoma cells (*n* = 3). (D) Analysis of caspase-3 enzyme activity (*n* = 5). (E) Western blot for quantifying apoptosis-related proteins (*n* = 3). (F) Western blot analysis showing the expression levels of EMT-related proteins in control and ZNF451-knockdown osteosarcoma cell lines (*n* = 3). (G) Transwell assay to measure cell migration and invasion (*n* = 3). Scale bar, 50 μm. (H and I) Flow cytometry for determining cell cycle (*n* = 3). Statistical significance denoted as *P < 0.05, **P < 0.01, and ***P < 0.001.

To investigate *ZNF451*’s function in osteosarcoma cells, we synthesized 3 targeted siRNAs against *ZNF451*, designated as si-ZNF451#1, si-ZNF451#2, and si-ZNF451#3, to examine *ZNF451*’s function in osteosarcoma cells. Our data demonstrated that all 3 siRNAs successfully decreased *ZNF451* expression, with si-ZNF451#1 being the most effective (*n* = 3; Fig. S11C). Consequently, si-ZNF451#1 was selected for further experiments involving *ZNF451* silencing. Following cisplatin treatment, we noted a marked reduction in cell viability and IC_50_ values in the si-*ZNF451*-treated group compared to the si-NC control group (*n* = 5; Fig. S11D and E). Both EdU assays (*n* = 3; Fig. 10A) and cell colony formation assays (*n* = 3; Fig. 10B) indicated that *ZNF451* suppression reduced the proliferation of MG63/R and U2OS/R cells. Furthermore, flow cytometry (*n* = 3), caspase-3 activity (*n* = 5), and Western blotting (*n* = 3) indicated an increase in apoptosis following *ZNF451* knockdown (Fig. 10C to E). In vitro experiments also revealed that *ZNF451* knockdown promoted EMT in drug-resistant osteosarcoma cell lines (*n* = 3; Fig. 10F). Additionally, transwell assays showed a significant reduction in the migratory and invasive abilities of MG63/R and U2OS/R cells after si-*ZNF451* treatment (*n* = 3; Fig. 10G). Flow cytometry analysis indicated that *ZNF451* knockdown significantly reduced the proportion of cells in the S phase and increased the number of cells in the G_2_-M phase. The knockdown did not significantly affect the proportion of cells in the G_1_-G_0_ phase in MG63/R cells, whereas in U2OS/R cells, there was a significant reduction in the number of cells in the G_1_-G_0_ phase (*n* = 3; Fig. 10H and I). Furthermore, we investigated whether *ZNF451* overexpression enhanced cisplatin resistance in MG63 and U2OS osteosarcoma cells. As shown in Fig. S11F, the overexpression of *ZNF451* in MG63 and U2OS cells enhanced their resistance to cisplatin. Following cisplatin treatment, both MG63 and U2OS cells overexpressing *ZNF451* exhibited significantly increased proliferation and reduced apoptosis compared to the control group (Fig. S11G to I). These results further support the notion that *ZNF451* enhances drug resistance in tumor cells.

### Investigating *ZNF451*’s function in osteosarcoma through in vivo models

**Fig. 11.**
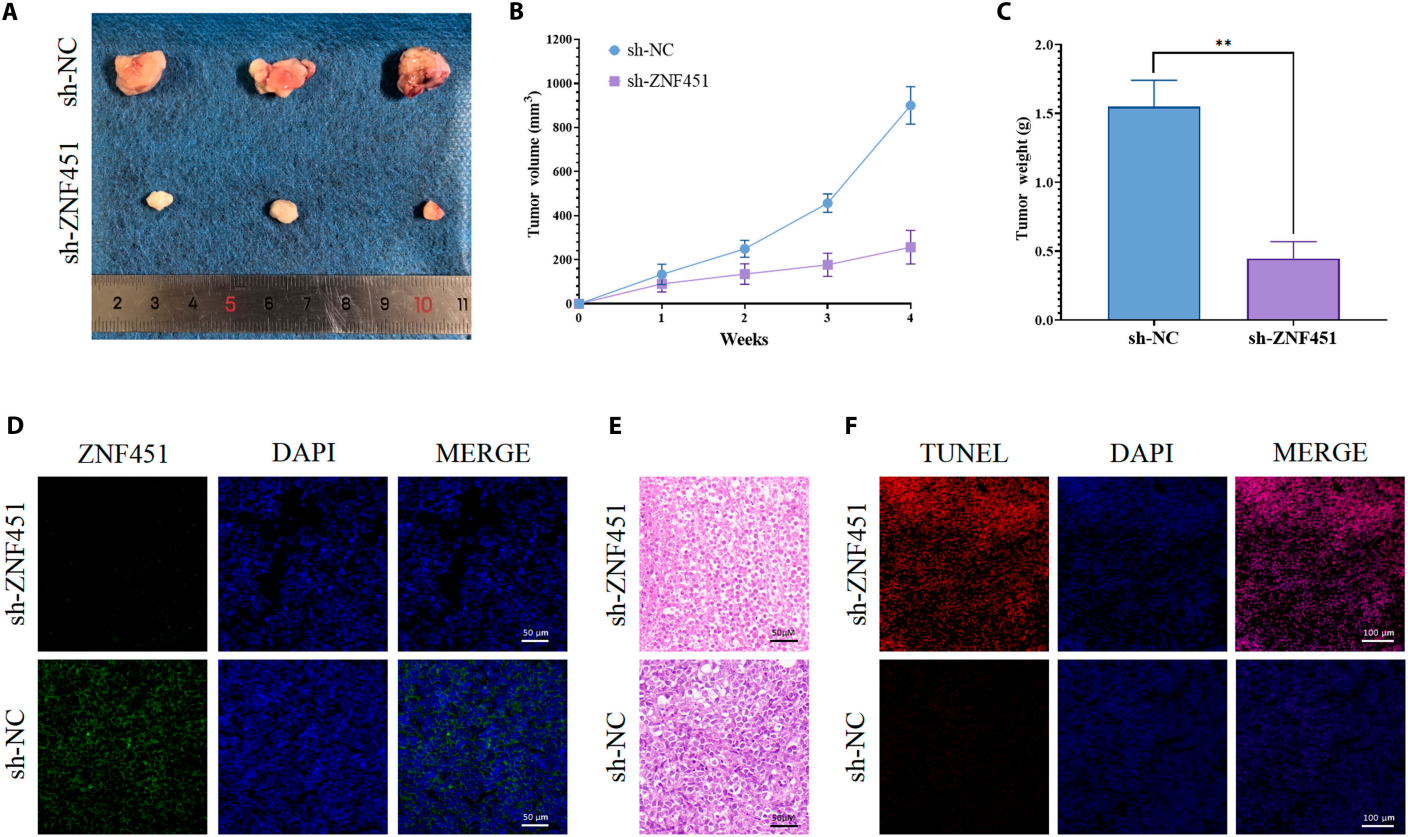
Exploration of ZNF451’s oncogenic potential in vivo. (A) Display of ex vivo tumor models from various experimental groups (*n* = 3). (B) Analysis of growth dynamics in ex vivo tumors among the groups (*n* = 3). (C) Weight comparison of ex vivo tumors from each group through statistical analysis (*n* = 3). (D) Immunofluorescence staining to detect ZNF451 expression in ex vivo tumor tissues (*n* = 3). Scale bar, 50 μm. (E) H&E staining of ex vivo tumor tissues (*n* = 3). Scale bar, 50 μm. (F) Evaluation of TUNEL-positive expression in ex vivo tumor tissues via immunofluorescence (*n* = 3). Scale bar, 100 μm.

We developed an animal model to assess *ZNF451*’s influence on tumor development. Tumor volume significantly increased in mice treated with sh-NC U2OS cells and decreased notably in mice treated with sh-*ZNF451*- U2OS (*n* = 3; Fig. 11A and B). As depicted in Fig. 11C (*n* = 3), the tumor mass was considerably reduced in the sh-*ZNF451* group compared to the control group. Immunofluorescence analysis revealed a substantial reduction in *ZNF451* protein expression in the sh-ZNF451-treated mice (*n* = 3; Fig. 11D). H&E staining provided evidence of a reduced tumor growth rate in the sh-ZNF451 group (*n* = 3; Fig. 11E). Furthermore, TUNEL staining showed increased apoptosis in the sh-ZNF451 group, especially in the heightened count of TUNEL-positive cells marked with red fluorescence (*n* = 3; Fig. 11F). Considering these findings, our results suggest that *ZNF451* silencing accelerates apoptosis in osteosarcoma cells and restricts their proliferation.

### Investigating *ZNF451*’s influence on bone resorption

**Fig. 12.**
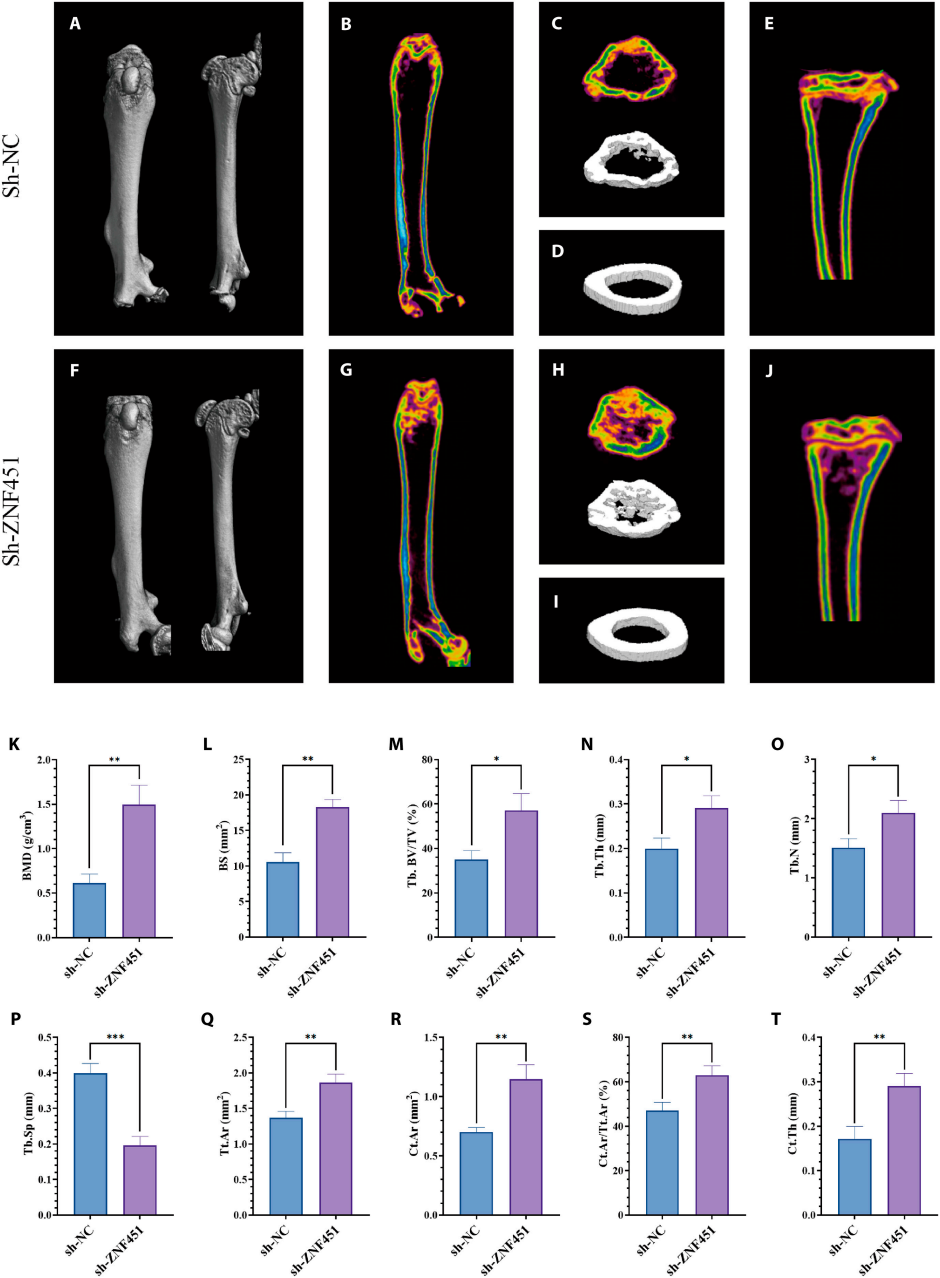
Micro-CT analysis of ZNF451 in osteosarcoma-related bone resorption. (A to E) Femoral 3D reconstructions in the sh-NC group (*n* = 3), including anterolateral (A), entire femur coronal cross-section (B), metaphyseal distal femur cross-section and 3D view (C), mid-femur cortical bone 3D view (D), and proximal tibia coronal view (E). (F to J) Similar femoral 3D reconstructions in the sh-ZNF451 group (*n* = 3), showing anterolateral (F), entire femur coronal section (G), metaphyseal distal femur cross-section, and 3D view (H), mid-femur cortical bone 3D view (I), and proximal tibia coronal view (J). (K to P) Trabecular bone microstructural parameter quantification (*n* = 3): BMD, BS , BV/TV, Tb.Th, Tb.N, Tb.Sp. (Q to T) Cortical bone microstructural parameter variations: Tt.Ar, Ct.Ar, Ct.Ar/Tt.Ar, Ct.Th. *P < 0.05, **P < 0.01, ***P < 0.001.

To determine *ZNF451*’s effect on the trabecular bone at the distal femoral metaphysis and the cortical bone at the midshaft of the femur, we utilized in vivo micro-computed tomography (CT) for 3D reconstruction of the femoral structure within the animal model (*n* = 3; Fig. 12A and F). Quantitative analysis of both trabecular and cortical bones revealed that, compared to the sh-NC group, the sh-*ZNF451* group exhibited a significant decrease in bone loss. Notably, bone mass increased in both trabecular and cortical regions in the sh-*ZNF451* group (Fig. 12B to D and G to I). Relative to the sh-NC group, the sh-*ZNF451* group exhibited marked increases in BMD, BS, BV/TV, Tb.Th, and Tb.N in the trabecular bone of the distal femoral metaphysis, along with a significant reduction in Tb.Sp (Fig. 12K to P). Furthermore, in the cortical bone at the midshaft of the femur, the sh-*ZNF451* group showed a considerable increase in Tt.Ar, Ct.Ar, Ct.Ar/Tt.Ar, and Ct.Th (Fig. 12Q to T). Additionally, an increase in bone mass was noted in the trabecular bone of the proximal tibia in the sh-*ZNF451* group compared to that in the sh-NC group (Fig. 12E and J).

### β-Cryptoxanthin may inhibit malignant behavior in cisplatin-resistant osteosarcoma cells by targeting *ZNF451*

**Fig. 13.**
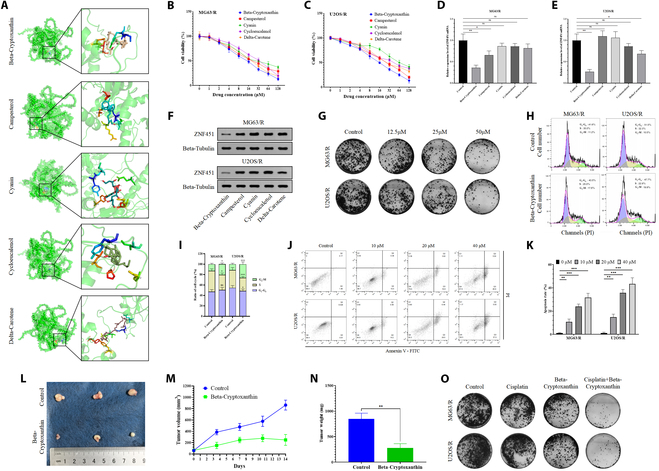
β-Cryptoxanthin suppresses the malignant characteristics of cisplatin-resistant osteosarcoma cells both in vitro and in vivo. (A) Docking outcomes of the TCM monomers with the lowest binding energies. (B and C) CCK-8 assay results demonstrate the effects of various concentrations of Chinese medicine monomers on the viability of MG63/R and U2OS/R cells (*n* = 5). (D to F) Measurement of ZNF451 mRNA levels [*n* = 3, (D) and (E)] and ZNF451 protein expression [*n* = 3, (F)] in MG63/R and U2OS/R cells treated with half-maximal inhibitory concentrations of TCM monomers. (G) Colony formation assay following treatment with different concentrations of TCM (*n* = 3). (H and I) Flow cytometry analysis of cell cycle phases in various treatment and control groups (*n* = 3). (J and K) Flow cytometry analysis of apoptosis in cells treated with different concentrations of TCM (*n* = 3). (L) Photographs of PDX tumors in NOD-SCID mice from the various treatment groups (*n* = 3). (M) Tumor growth curves of PDX tumors in NOD-SCID mice across different treatment groups (*n* = 3). (N) Tumor weight measurements of PDX tumors in NOD-SCID mice from the various treatment groups (*n* = 3). (O) Colony formation assay was used to detect the combined effect of drugs (*n* = 3). *P < 0.05, **P < 0.01, ***P < 0.001.

To identify potential traditional Chinese medicine (TCM) interactions with *ZNF451*, we used the Coremine Medical database (https://www.pubgene.com/coremine-medical/) to retrieve relevant information on this gene and identify significantly associated TCMs. We screened major active components of frequently identified TCMs (statistical frequency ≥2) using the TCM Systems Pharmacology Database and Analysis Platform (TCMSP), focusing on oral bioavailability (OB) ≥30% and drug-likeness (DL) ≥0.18. Molecular docking was performed to test the binding energies of all TCM monomers with *ZNF451*, with each monomer undergoing 3 independent docking experiments, and the results were visualized (Fig. S12). Finally, we identified the 5 TCM monomers with the lowest binding energies: β-cryptoxanthin, campesterol, cyanidin, loliolide, and δ-carotene (Fig. 13A). We treated MG63/R and U2OS/R cell lines with these monomers at different concentrations for 72 h and assessed the inhibition of cell proliferation using a CCK-8 assay. The results showed significant inhibitory effects on resistant osteosarcoma cells with increasing drug concentrations, with β-cryptoxanthin showing the most pronounced inhibition in both cell lines (*n* = 5; Fig. 13B and C). Subsequent experiments used the IC_50_ value of each monomer in subsequent experiments to explore their potential mechanisms of action in inhibiting tumor cell activity. Interestingly, qRT-PCR and Western blot analyses revealed that β-cryptoxanthin significantly inhibited *ZNF451* transcription and translation in MG63/R and U2OS/R cell lines, while other drugs had minimal effects on this gene (*n* = 3; Fig. 13D to F).

The colony formation assay showed a gradual decrease in cell proliferation with increasing concentrations of β-cryptoxanthin (*n* = 3; Fig. 13G). Treatment with IC_50_ concentrations of β-cryptoxanthin significantly arrested the cell cycle, with a notable increase in G_2_-M phase cells and a decrease in S and G_1_ phase cells (*n* = 3; Fig. 13H and I). Additionally, β-cryptoxanthin promoted apoptosis in a concentration-dependent manner in resistant cell lines (*n* = 3; Fig. 13J and K). Next, we established a patient-derived xenograft (PDX) mouse model. Once tumors reached an appropriate size, PDX mice were treated with β-cryptoxanthin. After 2 weeks of continuous treatment, β-cryptoxanthin significantly inhibited tumor growth compared to the control group (*n* = 3; Fig. 13L). Moreover, both tumor volume and weight were significantly reduced in the treatment group compared to the control group (*n* = 3; Fig. 13M and N). Cotreatment of MG63/R and U2OS/R cells with IC_50_ concentrations of β-cryptoxanthin and cisplatin significantly reduced the proliferation rate (*n* = 3; Fig. 13O). These findings suggest that β-cryptoxanthin may enhance the sensitivity of cisplatin-resistant osteosarcoma cell lines to cisplatin by targeting *ZNF451*.

## Discussion

Addressing recurrent osteosarcoma remains a significant challenge in the clinical setting [46,47]. Although advancements have been made in the treatment of localized osteosarcoma, recurrence typically results in reduced treatment efficacy and a corresponding deterioration in patient prognosis [48]. Tumor recurrence is closely linked to cellular drug resistance, complicating treatment [49,50]. Cisplatin, a standard chemotherapeutic agent for osteosarcoma, is often rendered less effective by the development of drug resistance, presenting a major hurdle to improving patient survival rates [51,52]. Several studies have explored the causes of cisplatin resistance and potential therapeutic approaches. For example, Wang et al. [53] revealed that the up-regulation of miR-1293 enhances cisplatin-induced apoptosis in osteosarcoma cells by inhibiting TIMP1 and its downstream Notch1/Hes1 and TGFBR1/Smad2/3 pathways. Tang et al. [54] found that Sestrin2 enhances chemotherapy resistance in osteosarcoma by enhancing autophagy and inhibiting apoptosis. Additionally, ursolic acid enhances cisplatin-induced DNA damage in osteosarcoma cells by promoting ferritinophagy and ferroptosis, thereby synergistically reducing drug resistance and inhibiting tumor growth. He et al. [55] explored an innovative strategy using targeted arsenene nanosheets (Her2-ANs@CDDP) to overcome cisplatin resistance in osteosarcoma cells. However, the specific mechanisms underlying cisplatin resistance are not fully understood. This underscores the urgent need for innovative treatment approaches and the identification of novel therapeutic targets to enhance patient survival rates and quality of life.

In the pursuit of new cancer treatments, the role of SUMOylation, a pivotal posttranslational modification process, is not well understood [56]. SUMOylation involves the covalent addition of SUMOs to specific proteins, which in turn affects their function, distribution, and stability [41,42]. This process is instrumental in key cellular functions, such as the regulation of gene expression, DNA repair, and cell cycle governance [57]. During cancer progression, aberrant SUMOylation patterns, resulting from either overactivation or malfunction, are intricately linked to tumor initiation, growth, aggression, recurrence, and metastasis. Therefore, targeting abnormal SUMOylation has emerged as a crucial area of cancer research and presents novel opportunities for therapeutic intervention [58].

With the support of single-cell technologies, researchers can analyze tumor heterogeneity and complex intercellular interactions within the tumor microenvironment with unprecedented resolution [59]. This technology allows for the in-depth exploration of gene expression differences among cell populations, revealing the specific roles of different cell types in disease progression and identifying potential drug targets. For example, Liu et al. [60] revealed the synergistic effects of 11-keto-β-boswellic acid and Z-guggulsterone in treating ischemic stroke using single-cell transcriptomics, identifying Spp1 as a key target of KBA-Z-GS. By analyzing multiple single-cell and bulk sequencing datasets, we compared primary and recurrent osteosarcomas, revealing a significant up-regulation of divergent signaling pathways in recurrent osteosarcomas, particularly those associated with SUMOylation. Through an in-depth analysis of osteosarcoma samples using single-cell data, we identified dysregulation or functional abnormalities of the SUMOylation-related gene *ZNF451*, which correlated positively to the recurrence and prognosis of osteosarcoma. *ZNF451* is a zinc-finger domain-containing protein belonging to the SUMO E3 ligase family [61]. It was initially discovered in the promyelocytic leukemia (PML) protein nuclear bodies [61–63]. Multiple studies have shown that *ZNF451* regulates protein stability through SUMOylation, thereby promoting tumor progression [64,65]. For instance, *ZNF451* interacts with SLUG, facilitating SLUG-mediated CCL5 transcription, thereby driving the development of triple-negative breast cancer [65]. By employing enrichment analysis methods such as GO, KEGG, GSVA, and GSEA, we found that the gene is enriched in multiple pathways related to cancer progression, suggesting that *ZNF451* might play a crucial role in reshaping the tumor immune microenvironment and cancer development. These enrichment results are consistent with those of previous studies on *ZNF451* [64–66].

In both in vivo and in vitro analyses, we explored the effects of *ZNF451* on osteosarcoma development and progression. Our findings revealed that *ZNF451* is overexpressed in osteosarcoma cell lines, with an even more pronounced increase in cisplatin-resistant variants. Previous studies have suggested that *ZNF451*-mediated TOP2cc repair pathway may help tumor cells adapt to treatment with TOP2 inhibitors during chemotherapy [66,67]. This adaptation can lead to tumor cell resistance to chemotherapeutic drugs and can indirectly promote cancer progression. Overexpression of *ZNF451* in cisplatin-resistant osteosarcoma cell lines may be associated with enhanced tumor drug resistance.

Apoptosis plays a crucial role in increasing chemosensitivity [53]. Previous studies have shown that *ZNF451* inhibits apoptosis of human pancreatic ductal adenocarcinoma cells [64]. In this study, we found that *ZNF451* reduced the sensitivity of parental osteosarcoma cell lines to cisplatin, thereby inhibiting apoptosis. In contrast, *ZNF451* knockout in cisplatin-resistant osteosarcoma cell lines significantly increased apoptosis. Moreover, *ZNF451* stabilizes TWIST2 via SUMOylation, which promotes EMT [68]. In this study, we found that *ZNF451* knockout significantly reduced N-cadherin expression and increased E-cadherin expression in resistant osteosarcoma cell lines, leading to enhanced cell adhesion. Additionally, the *ZNF451* knockout significantly weakened the migration and invasion abilities of these cells. Studies have indicated that N-cadherin acts as a tumor suppressor in osteosarcoma cells, with its reduced expression typically associated with decreased cell adhesion [53,69]. Lower cell adhesion improves drug permeability, thereby increasing sensitivity to chemotherapy [53,70]. Therefore, our findings support the mechanism by which *ZNF451* regulates the expression of cell adhesion-related proteins, weakens cell adhesion, promotes cell migration and invasion, and reduces sensitivity to cisplatin. Collectively, these results reveal the key role of *ZNF451* in regulating osteosarcoma cell sensitivity to chemotherapeutic drugs.

Osteosarcoma cells secrete factors that promote bone resorption, stimulating osteoclast differentiation and activity, which, in turn, drives bone dissolution and facilitates tumor growth, invasion, and distant metastasis [71]. These cells promote osteoclast differentiation and activation by secreting specific osteoclast-stimulating factors such as RANKL, further accelerating bone tissue breakdown [71–74]. According to the micro-CT analysis, silencing of *ZNF451* demonstrated notable anti-resorptive effects. This was evidenced by significant increases in BMD, bone surface area, BV/TV, and Tb.Th, Tb.N, Tt.Ar, Ct.Ar, Ct.Ar/Tt.Ar, and Ct.Th, along with a marked reduction in Tb.Sp. These results underscore the crucial role of *ZNF451* in sustaining osteosarcoma malignancy and its potential as a predictive biomarker for the biological behavior and progression of the disease.

TCM is considered a valuable source of candidate small-molecule drugs because of their diverse bioactivities [75]. Many natural products employed in TCM exhibit significant anticancer effects, including the inhibition of tumor proliferation and angiogenesis, induction of apoptosis, modulation of autophagy, reversal of multidrug resistance, regulation of immune balance, and enhancement of chemotherapy efficacy [76]. The antitumor effects of β-cryptoxanthin are primarily attributed to its antioxidant and antiproliferative properties [77,78]. Studies have shown that β-cryptoxanthin induces apoptosis and inhibits the proliferation and migration of cancer cells by modulating various cell signaling pathways [32]. Additionally, β-cryptoxanthin has demonstrated significant anticancer effects in animal models [34,79]. However, its role in osteosarcoma remains unclear. Therefore, we investigated its effects on cisplatin-resistant osteosarcoma cells.

Our study demonstrated that β-cryptoxanthin significantly reduced the expression level of *ZNF451*, thereby inhibiting the malignant phenotype of cisplatin-resistant osteosarcoma cells. Furthermore, β-cryptoxanthin treatment not only reduced cell proliferation and caused G_2_-M phase cell cycle arrest but also induced apoptosis in resistant osteosarcoma cell lines. These in vitro findings were validated in a PDX mouse model, where β-cryptoxanthin significantly inhibited tumor growth. Additionally, β-cryptoxanthin synergized with cisplatin, enhancing the sensitivity of resistant osteosarcoma cell lines to cisplatin. These results suggest that β-cryptoxanthin, by regulating *ZNF451* expression, not only inhibits the malignant behavior of cisplatin-resistant osteosarcoma cells but also increases their sensitivity to cisplatin, highlighting its potential application in osteosarcoma treatment.

CD8^+^ T cells, which are crucial effectors in the immune response, specifically target and eliminate cells [80]. However, within the intricate immune microenvironment of tumors, tumor-directed CD8^+^ T cells often undergo functional exhaustion, which is characterized by diminished effector functionality and reduced proliferation [81,82]. This state is important in the progression of osteosarcoma and treatment resistance. In our study, we utilized pseudo-temporal analysis to investigate the dynamic shifts in CD8^+^ T cells and identified significant up-regulation of *ZNF451* in CD8^+^ TEXH cells. This observation implies that *ZNF451* may be involved in the transition of naive CD8^+^ T cells to CD8^+^ TEXH cells, thereby affecting the evolving immune microenvironment in osteosarcoma. To validate the results of the bioinformatics analysis, we employed experimental methods to demonstrate that *ZNF451* is highly expressed in exhausted CD8^+^ T cells within tumor tissues. More importantly, our experiments demonstrated that *ZNF451* not only exhibits high expression in these cells but also regulates PD1 expression in CD8^+^ T cells, thereby inducing their exhaustion and playing a key role in promoting this process. These findings further enhance our understanding of the role of *ZNF451* in the tumor immune microenvironment, suggesting that *ZNF451* may influence tumor immune evasion and the development of therapeutic resistance by regulating the exhaustion of CD8^+^ T cells.

*ZNF451*, a key molecule, shows great potential for clinical applications in osteosarcoma. First, high *ZNF451* expression is closely related to chemotherapy resistance and could serve as an important biomarker for predicting treatment responses. These findings may assist clinicians in formulating personalized treatment plans based on *ZNF451* expression levels, particularly for managing drug-resistant osteosarcoma. Second, *ZNF451* holds potential as a therapeutic target for reversing tumor cell resistance and enhancing the efficacy of chemotherapy. Notably, β-cryptoxanthin has shown the potential to down-regulate *ZNF451*, making it a promising candidate for targeted interventions. Targeting *ZNF451* with β-cryptoxanthin may offer a novel therapeutic approach to overcome cisplatin resistance, bringing hope to patients with cisplatin-resistant osteosarcoma. As further research continues to explore the function and mechanisms of *ZNF451*, this molecule is expected to play a significant role in the personalized treatment of osteosarcoma, offering new hope for improving patient outcomes.

Despite the significant findings of our study, several limitations remain. First, although advanced technologies such as single-cell sequencing and large-scale RNA-seq were employed, there is still room for improvement in the breadth and depth of the analysis. Second, while the study identified the critical role of ZNF451 in osteosarcoma progression and drug resistance, the precise mechanisms by which ZNF451 contributes to tumor resistance are not yet fully understood. Although we observed that ZNF451 inhibits apoptosis and promotes proliferation of osteosarcoma cells both in vitro and in vivo, the specific molecular pathways or signaling networks through which it induces resistance require further investigation. Future research should explore the interaction between ZNF451 and other known resistance, anti-apoptotic, and proliferative mechanisms to clarify its exact role in the development of tumor resistance and progression, which will provide a theoretical basis for new therapeutic strategies. Additionally, the mechanisms by which ZNF451 induces osteolysis remain unclear. While this study found that ZNF451 is associated with osteolysis, further investigation is needed to determine how it drives this process by regulating the balance between bone resorption and formation. Elucidating the role of ZNF451 in osteolysis will shed light on the biological basis of bone destruction in osteosarcoma patients and offer new therapeutic targets for clinical intervention. We also found that ZNF451 promotes CD8^+^ T cell exhaustion by inducing PDL1 expression, but the exact mechanism by which it drives the conversion of CD8^+^ T cells into an exhausted phenotype remains unclear and requires further investigation. Finally, considering the clinical translational potential of our findings, further drug optimization and experimental validation of β-cryptoxanthin are needed to ensure its safety and efficacy.

## Conclusion

This study elucidates the critical role of *ZNF451* in osteosarcoma cells. *ZNF451* significantly enhanced the growth, migration, and invasion abilities of resistant cells while reducing their sensitivity to cisplatin and apoptosis rates. Moreover, *ZNF451* plays a crucial role in promoting osteolysis, contributing to tumor progression through bone resorption. Our research also indicated that *ZNF451* regulates CD8^+^ T cell function, leading to their exhaustion and transition to the CD8T.EXH state, thereby disrupting the immune balance in the osteosarcoma microenvironment. Concurrently, β-cryptoxanthin may inhibit the malignant phenotype of cisplatin-resistant osteosarcoma cells by down-regulating *ZNF451* expression. Drug resistance in osteosarcoma cells results from interactions between multiple molecular mechanisms and pathways. Further comprehensive research is required to fully understand the effect of *ZNF451* on osteosarcoma resistance. Such studies are crucial for identifying new therapeutic targets and providing a solid scientific basis for the development of effective treatments against osteosarcoma resistance. Future research should aim to elucidate the complex interactions between *ZNF451* and osteosarcoma cells, providing deeper insights that will enable optimization of clinical treatment strategies.

## Data Availability

Single-cell data were retrieved from the GEO database (GSE152048, GSE162454, GSE198896, GSE169396, GSE217792), and bulk data were obtained from the GSE21257 database.
