# Supplementary material for: Single-Cell Spatial–Temporal Analysis of ZNF451 in Mediating Drug Resistance and CD8+ T Cell Dysfunction
Source: Research (Wash D C). 2024 Nov 12;7:0530. doi: 10.34133/research.0530 (PMC11555180; doi:10.34133/research.0530)
Supplement: Supplementary 1 — Figs. S1 to S12 Table S1 [file research.0530.f1.docx]

**
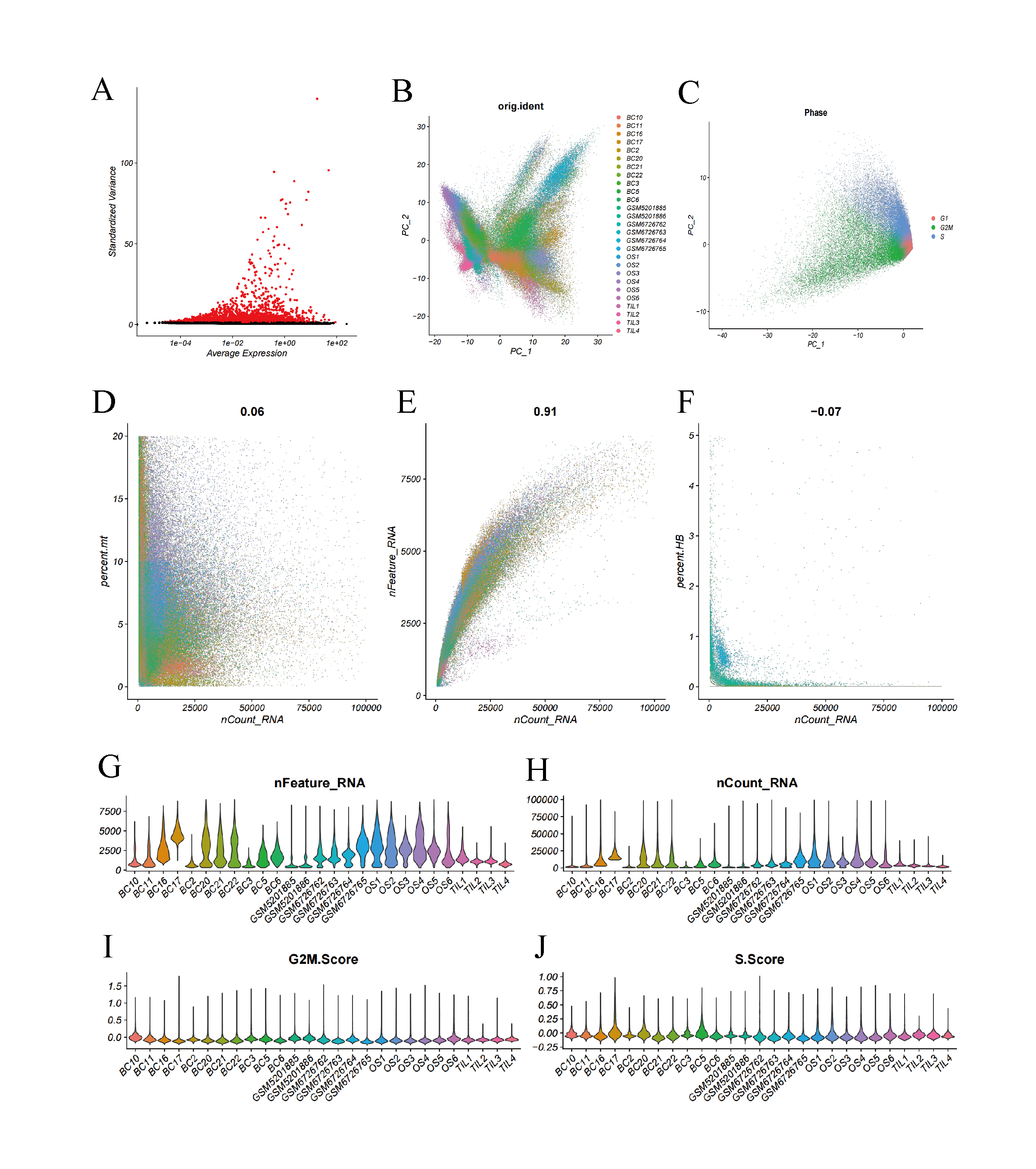
**

**Figure S1. Data Processing for scRNA-seq.** A. Assessment of gene variability within cells. B. Principal Component Analysis (PCA) applied to each individual sample. C. Evaluation of cell cycle phases using PCA. D-F. Correlation scatter plots depicting the relationship between total gene expression per cell and mitochondrial gene counts (D), total gene counts (E), and red blood cell gene count percentages (F). G. Statistical analysis of gene profiles in the samples. H. Comprehensive gene expression overview for all samples. I. Scoring of the G2/M phase transition. J. Scoring for the synthesis phase of the cell cycle.


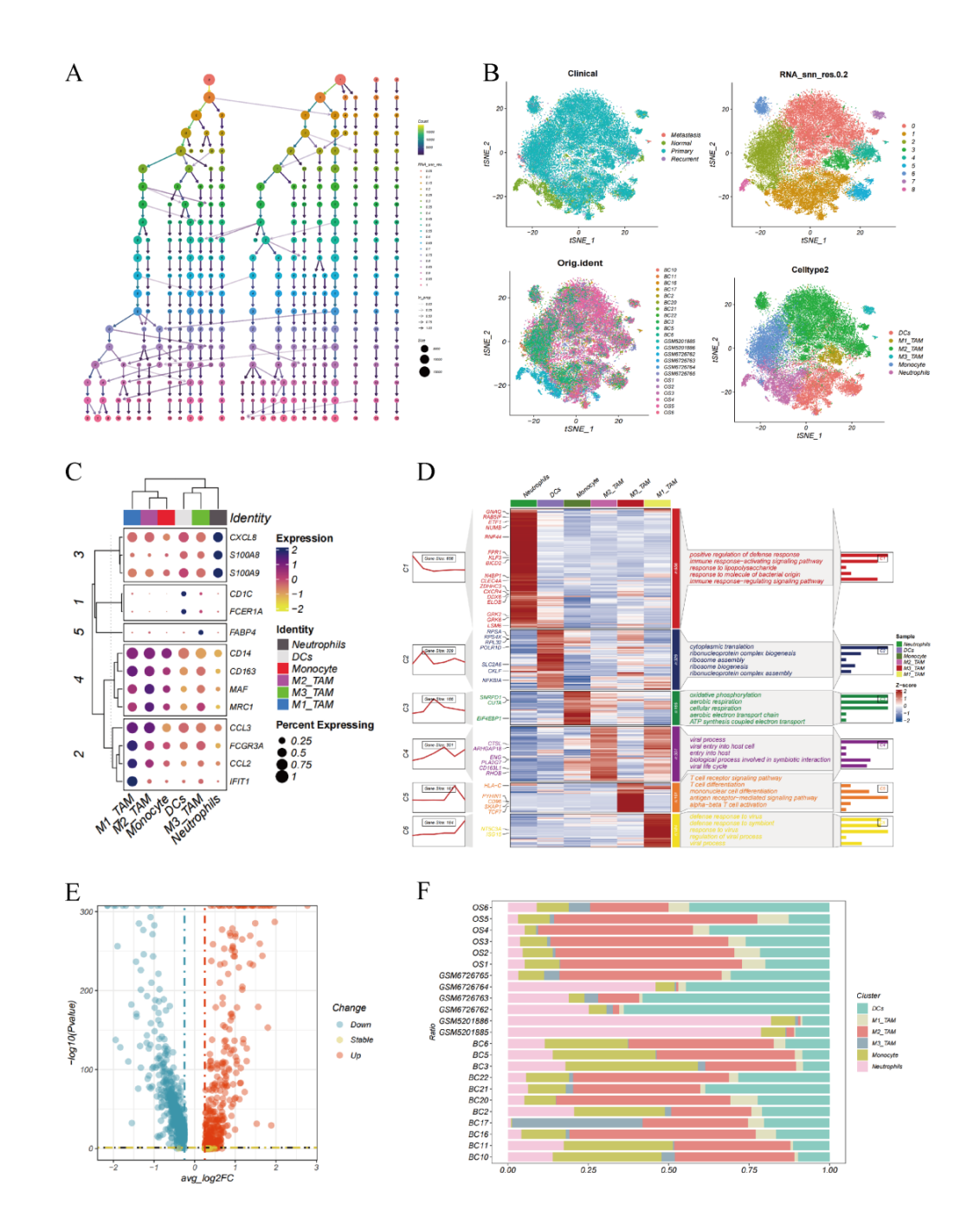


**Figure S2. Comprehensive Analysis of Myeloid Cells within the Immune Microenvironment.** A. Cell classification outcomes across various resolution levels. B. tSNE-visualized distribution of distinct clinical types, cell populations, samples, and cell categories. C. Identification of signature genes in specific myeloid cells. D. Heatmap and Gene Ontology (GO) analyses of myeloid cell genes exhibiting differential expression. E. Comparative gene expression analysis in myeloid cells from recurrent versus primary osteosarcomas, highlighting genes upregulated in recurrent cases (denoted in red). F. Proportional distributions of myeloid cell subpopulations across different samples.


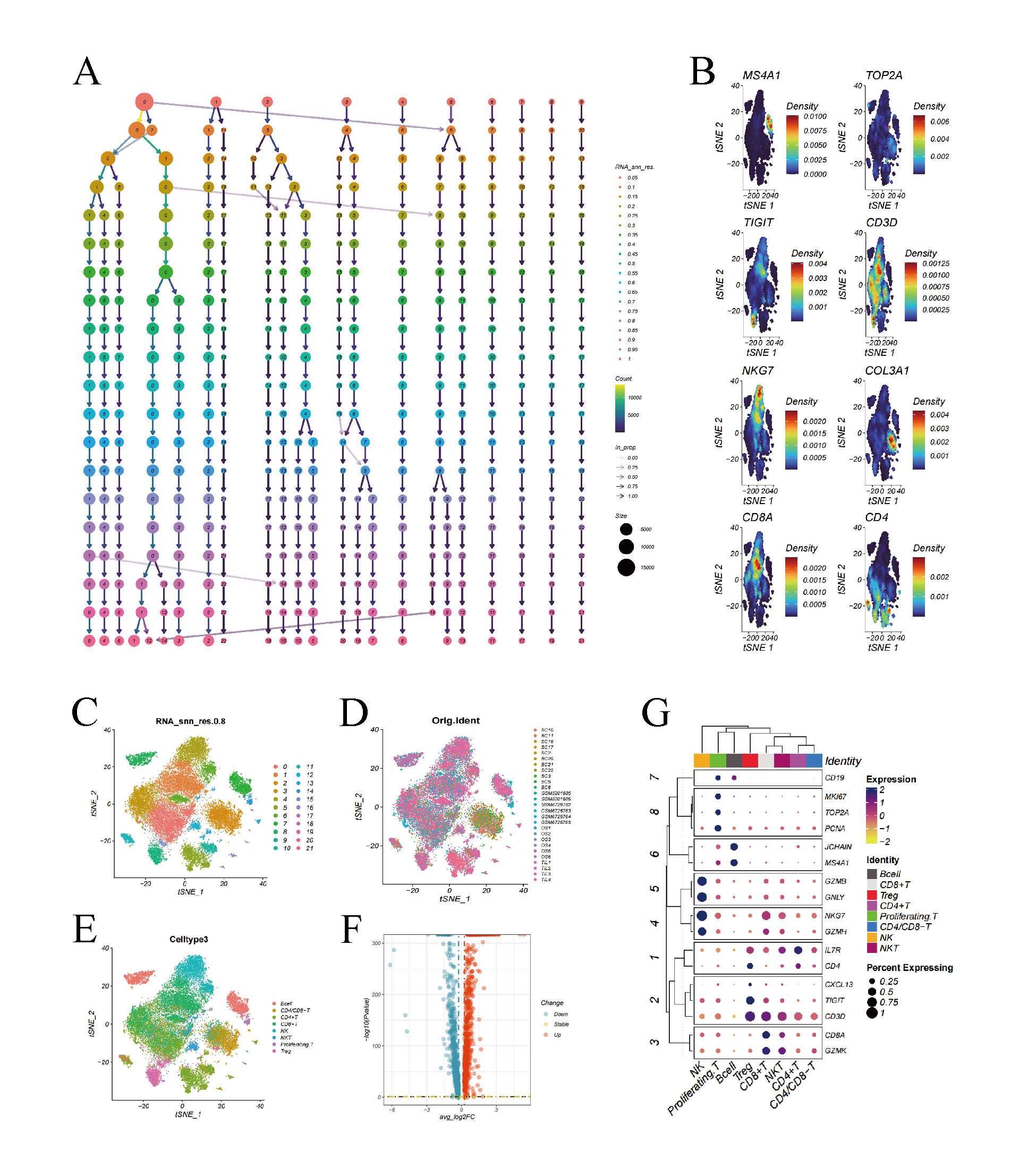


**Figure S3. In-Depth Analysis of Tumor-Infiltrating Lymphocytes (TILs) within the Tumor Immune Microenvironment.** A. Presentation of cell classification outcomes at various resolutions. B. UMAP visualization of marker genes associated with classical TILs. C-E. tSNE plot illustrating the distribution of cell populations, samples, and cell types. F. Volcano plot of differentially expressed genes (DEGs) in TILs from primary and recurrent osteosarcomas, highlighting genes upregulated in recurrent cases (shown in red). G. Identification of marker genes linked to classical TILs.

**
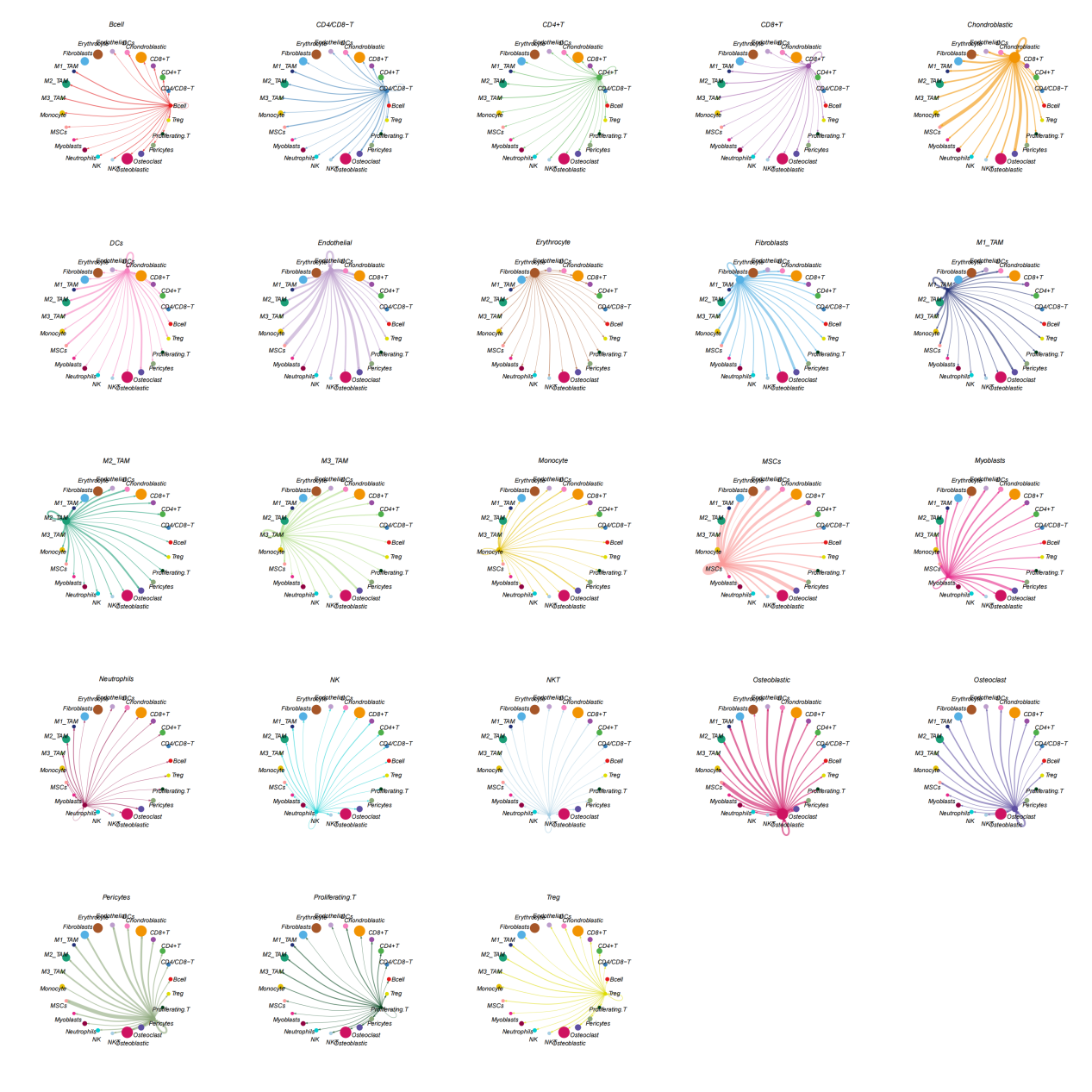
**

**Figure S4. Quantitative Depiction of Signal Emission Across Different Cell Subpopulations.**


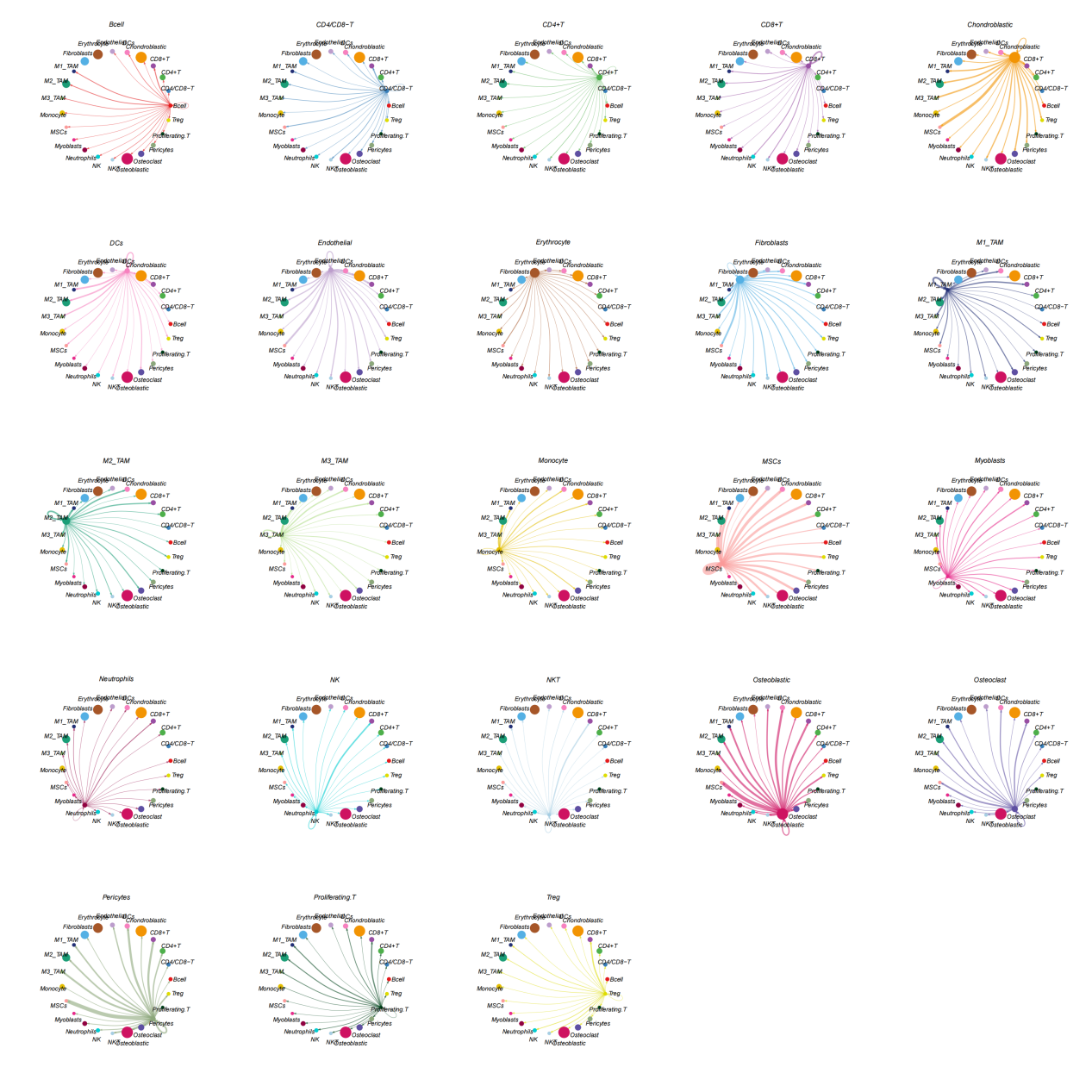


**Figure S5. Representation of Signal Intensity Variations Among Different Cell Subgroups.**


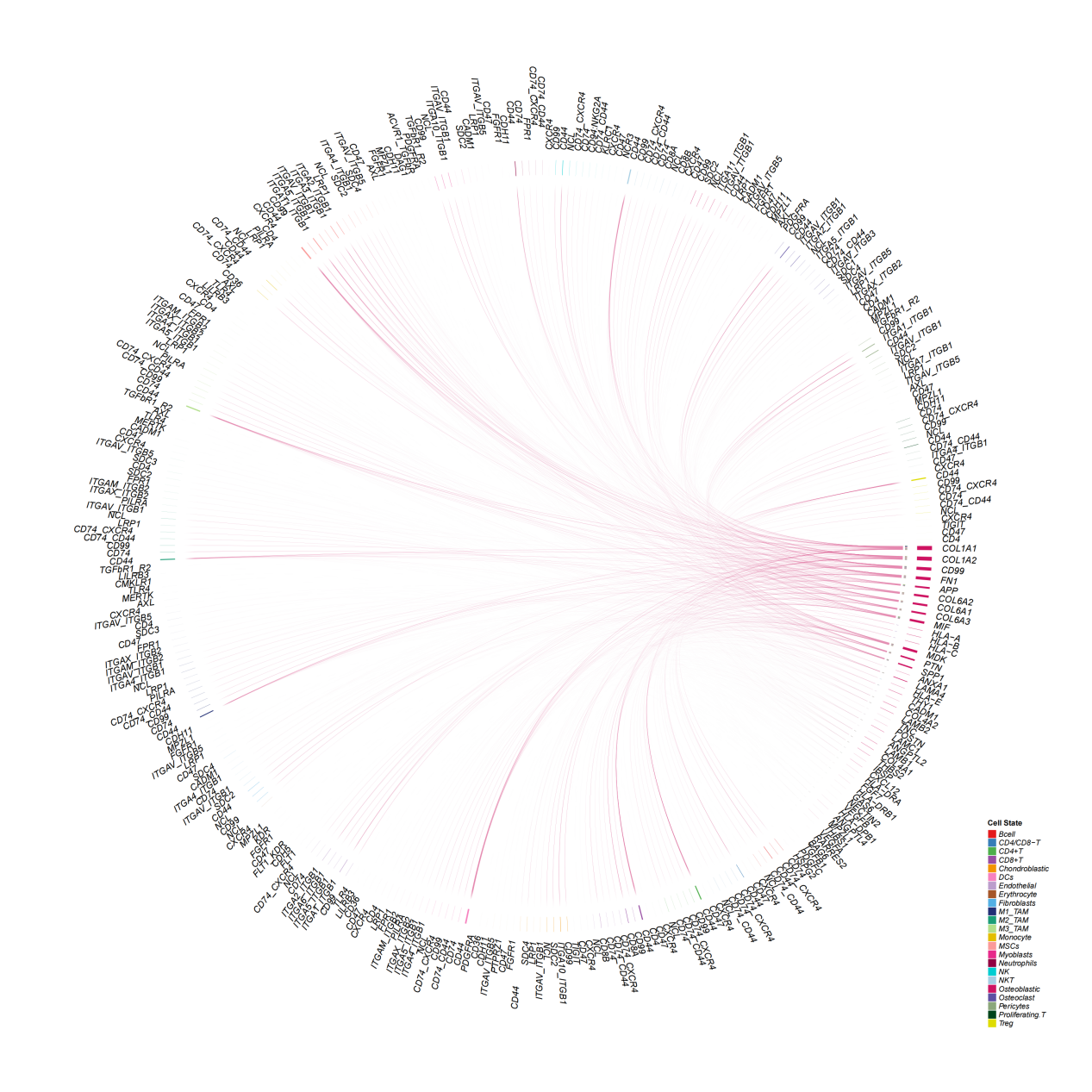


**Figure S6. Interaction of Osteoblasts with Diverse Cell Subpopulations Through Various Signaling Channels.**

**
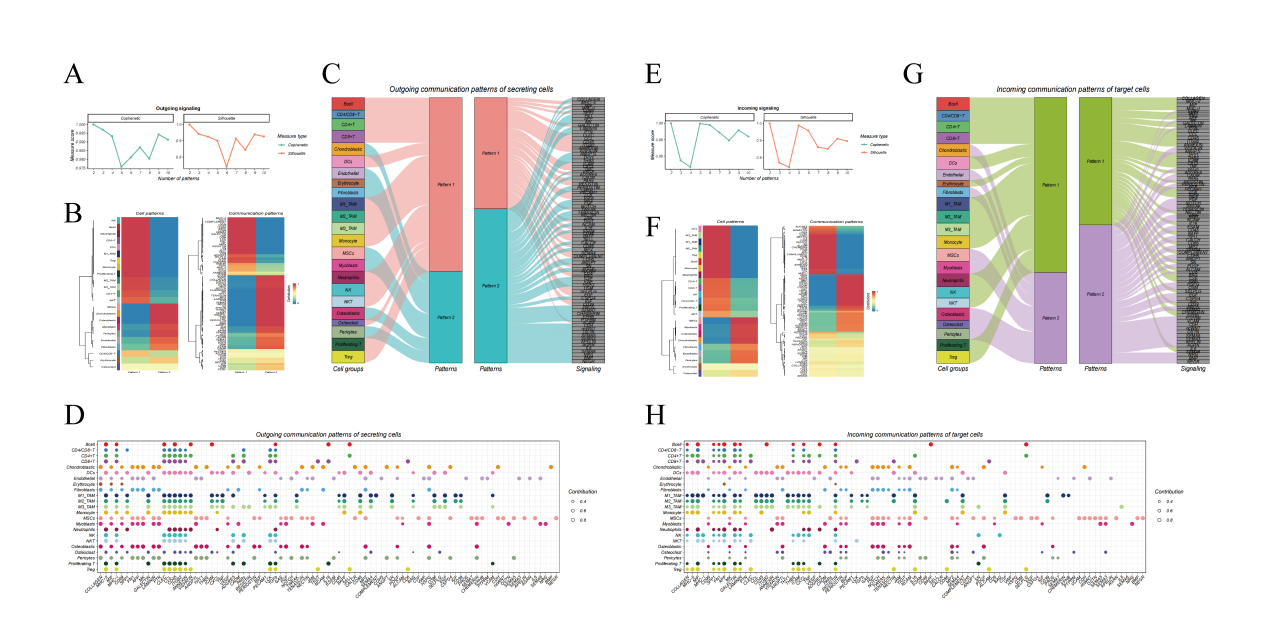
**

**Figure S7. Comprehensive Examination of Global Cellular Communication Networks.** A. Determination of output pattern quantities using cophenetic and silhouette metrics. B. Recognition of various cell subpopulations and their respective output signaling patterns. C. Sankey diagram illustrating the distribution of outgoing signal patterns from signal-emitting cells. D. Detailed plot showing variations in outgoing signals across key pathways. E. Calculation of input pattern numbers through cophenetic and silhouette evaluations. F. Identification of cell subgroups and their specific input signal patterns. G. Sankey diagram depicting the flow of incoming signal patterns to recipient cells. H. Detailed analysis of incoming signals within critical pathways.

**
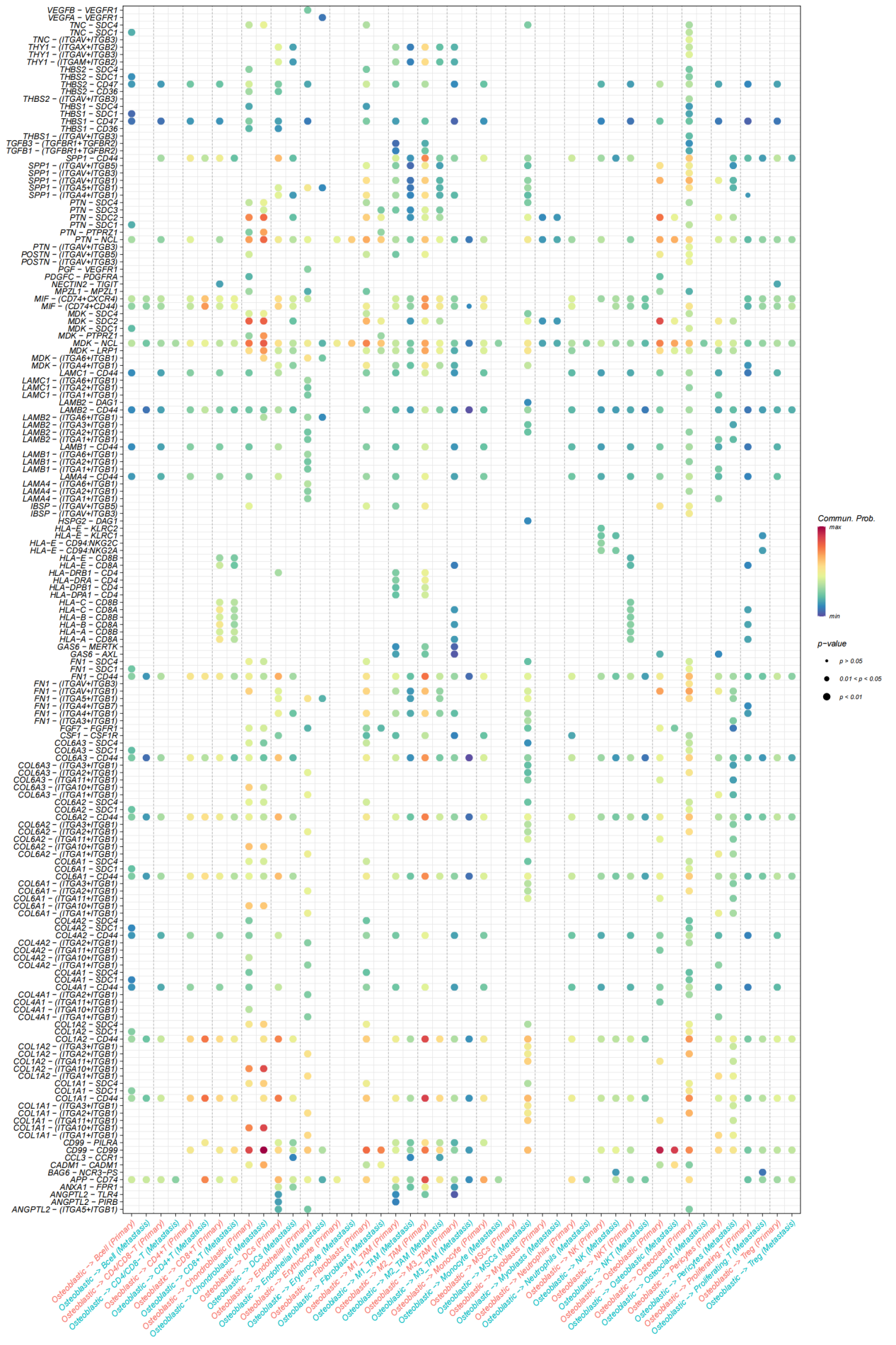
**

**Figure S8. Probabilistic Communication Modulation via Ligand-Receptor Interactions Originating from Osteoblasts or Chondroblasts to Other Cellular Congregates.**

**
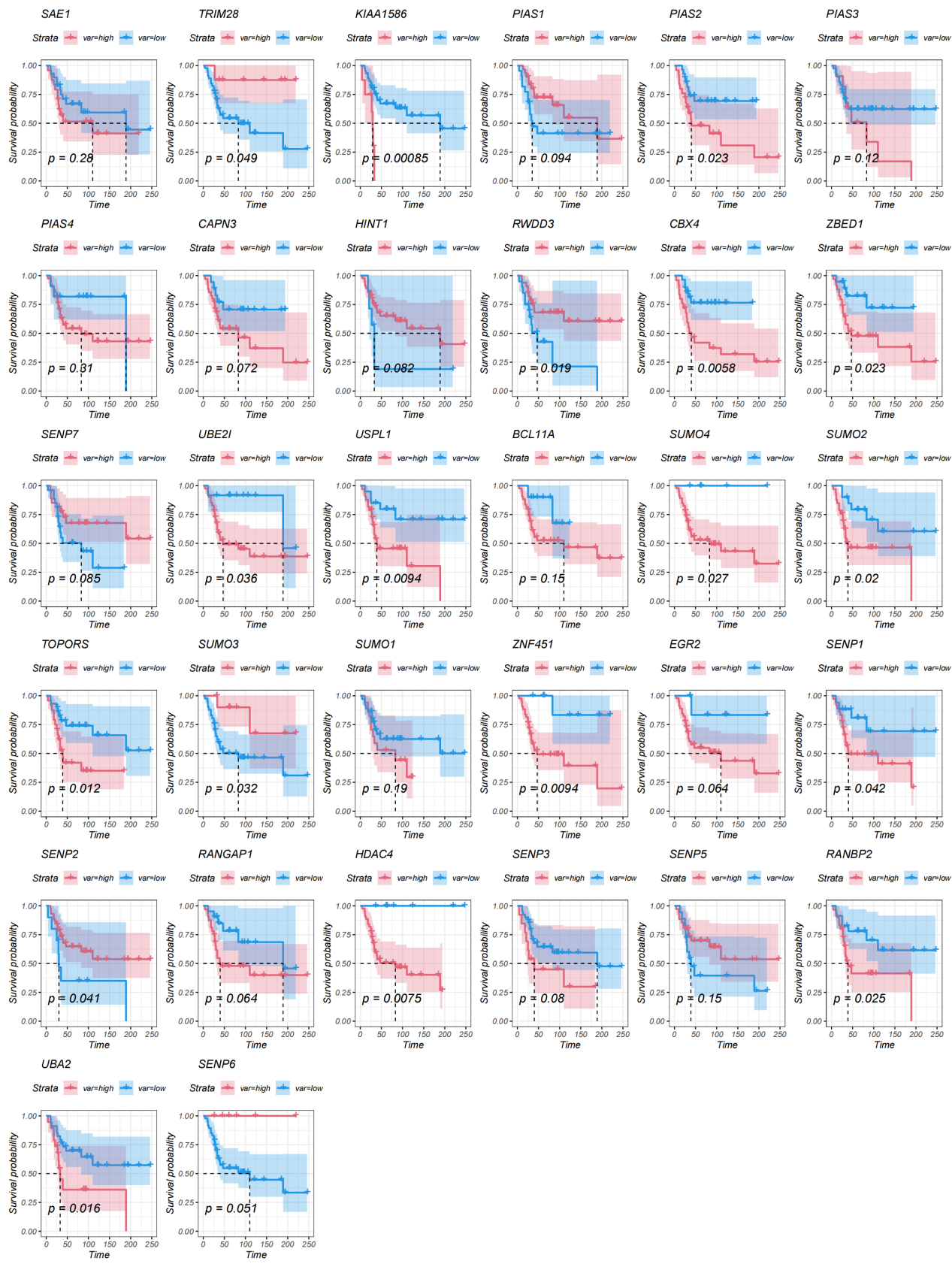
**

**Figure S9. Prognostic Assessment of OS-SUMOs Using Data from the GSE21257 Dataset.**

**
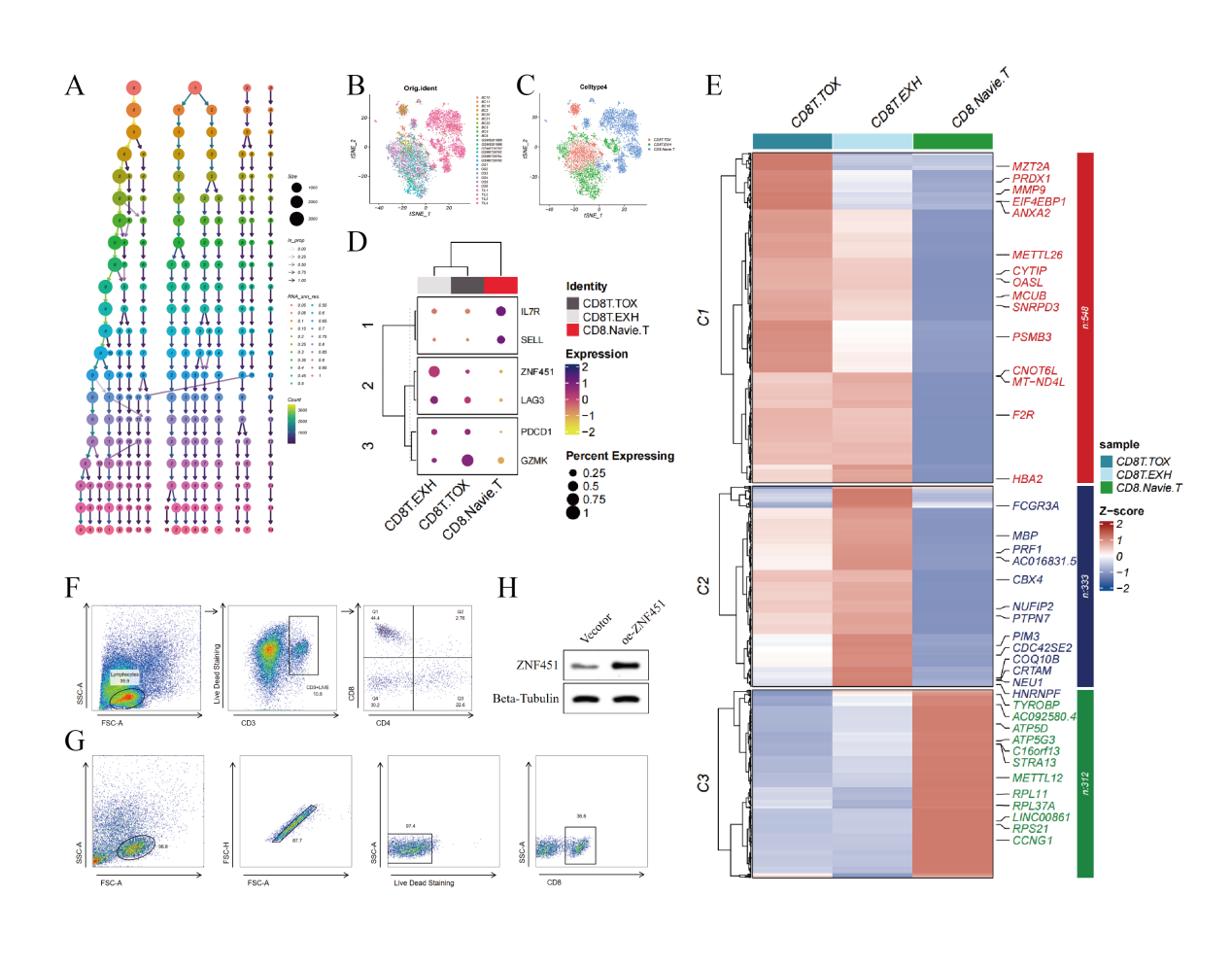
**

**Figure S10. Comprehensive Characterization and Recognition of CD8+ T Cell Variants.** A. Cell grouping at different resolution levels. B-C. tSNE mappings showcasing the diversity in CD8+ T cells based on sample sources (B) and cell phenotype (C). D. Spatial gene marker distribution charts for CD8+ T cell subgroups. E. Expression pattern heatmaps for genes within distinct CD8+ T cell subsets. F. Flow cytometry sorting of CD8+ T cells from osteosarcoma-infiltrating lymphocytes. G. Flow cytometry sorting of CD8+ T cells from peripheral blood. H. Western blot analysis of ZNF451 overexpression in CD8+ T cells.


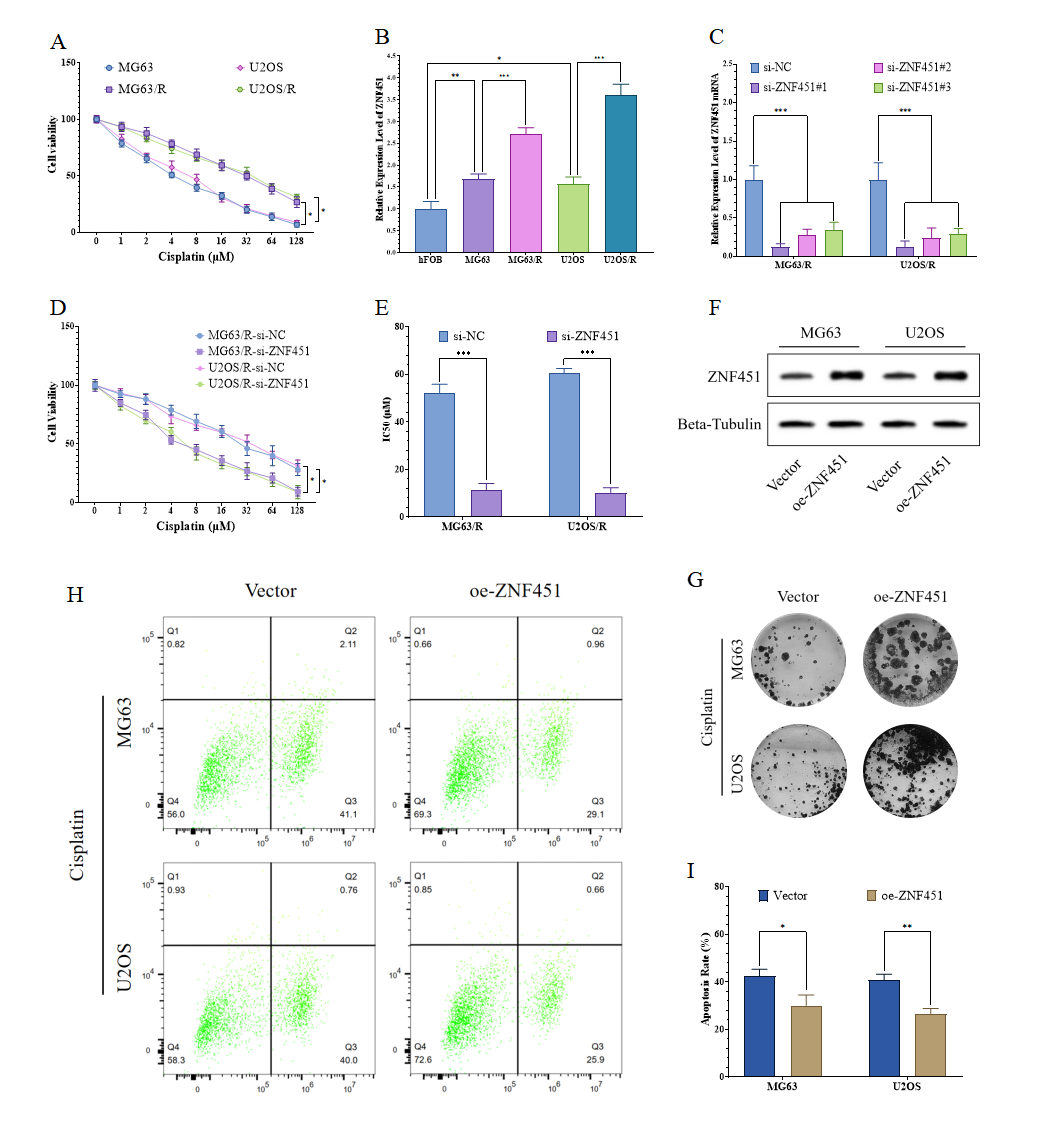


**Figure S11.** A. CCK-8 assays were performed to compare the osteosarcoma drug-resistant cell lines with their respective parental lines (n=5). B. Semi-quantitative analysis of Figure 9D was conducted using Image J software (n=3). C. qRT-PCR was used to assess the efficiency of ZNF451 knockdown post-siRNA treatment in osteosarcoma cells (n=3). D. Cell viability was evaluated using CCK-8 assays in osteosarcoma cells treated with varying cisplatin concentrations (n=5). E. The IC50 values were determined via CCK-8 assays for the various osteosarcoma cell groups (n=5). F. WB was used to assess the efficiency of ZNF451 overexpression in MG63 and U2OS cells (n=3). G. Colony formation assay (n=3). H-I. Flow cytometry for determining apoptosis rates in MG63 and U2OS cells (n=3). Statistical significance is denoted as follows: *P < 0.05, **P < 0.01, ***P < 0.001.


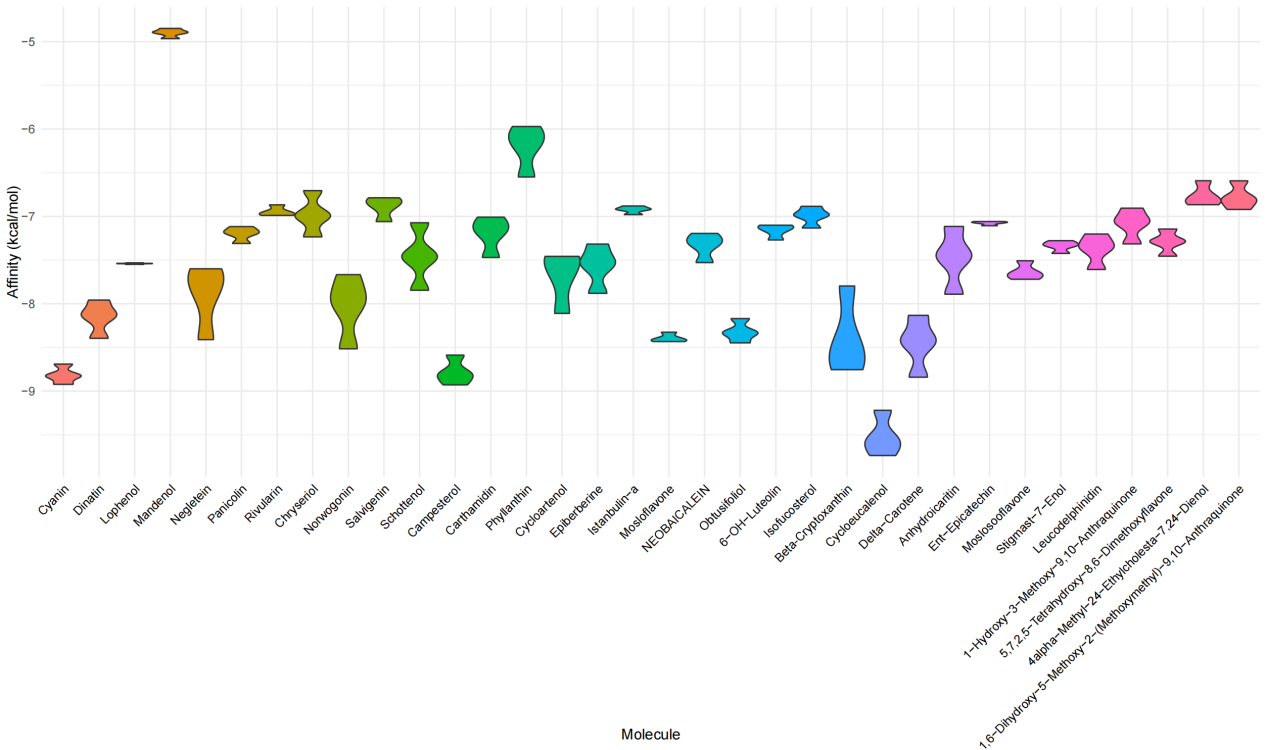


**Figure S12.** Binding energy results of molecular docking for 34 traditional Chinese medicine monomers (n=3).

**Table S1. Primers used for shRNA and siRNA**

| **shRNA OR siRNA** | **Primer sequences** |
| --- | --- |
| si- ZNF451#1 | GGUCAGCAGUGAUGAUGAAGA |
| si- ZNF451#2 | CGCUCAACUGUAAGAUUUAUA |
| si- ZNF451#3 | GAUGUUCCCUUUCAAGUUAAG |
| sh- ZNF451 | CCTTTGCTTGTGTAGTATGTT |
